# *In silico* approaches supporting drug repurposing for Leishmaniasis: a scoping review

**DOI:** 10.17179/excli2024-7552

**Published:** 2024-09-03

**Authors:** Gustavo Scheiffer, Karime Zeraik Abdalla Domingues, Daniela Gorski, Alexandre de Fátima Cobre, Raul Edison Luna Lazo, Helena Hiemisch Lobo Borba, Luana Mota Ferreira, Roberto Pontarolo

**Affiliations:** 1 Postgraduate Program in Pharmaceutical Sciences, Department of Pharmacy, Federal University of Paraná, Curitiba 80210-170, Paraná, Brazil

**Keywords:** neglected tropical diseases, computer-aided drug design, repositioning, docking, genomics, molecular dynamics

## Abstract

The shortage of treatment options for leishmaniasis, especially those easy to administer and viable for deployment in the world's poorest regions, highlights the importance of employing these strategies to cost-effectively investigate repurposing candidates. This scoping review aims to map the studies using *in silico* methodologies for drug repurposing against leishmaniasis. This study followed JBI recommendations for scoping reviews. Articles were searched on PubMed, Scopus, and Web of Science databases using keywords related to leishmaniasis and *in silico* methods for drug discovery, without publication date restrictions. The selection was based on primary studies involving computational methods for antileishmanial drug repurposing. Information about methodologies, obtained data, and outcomes were extracted. After the full-text appraisal, 34 studies were included in this review. Molecular docking was the preferred method for evaluating repurposing candidates (n=25). Studies reported 154 unique ligands and 72 different targets, sterol 14-alpha demethylase and trypanothione reductase being the most frequently reported. *In silico* screening was able to correctly pinpoint some known active pharmaceutical classes and propose previously untested drugs. Fifteen drugs investigated *in silico* exhibited low micromolar inhibition (IC_50_ < 10 µM) of *Leishmania *spp. *in vitro*. In conclusion, several *in silico* repurposing candidates are yet to be investigated *in vitro* and *in vivo*. Future research could expand the number of targets screened and employ advanced methods to optimize drug selection, offering new starting points for treatment development.

See also the graphical abstract(Fig. 1).

## Introduction

Leishmaniasis is a neglected tropical parasitic disease (NTD) caused by protozoans from the genus *Leishmania*. According to estimates from the World Health Organization (WHO), over one million new cases occur every year, particularly in the poorest regions of the world. The 2022 global surveillance data from WHO indicates that 99 countries are currently endemic for leishmaniasis (Ruiz-Postigo et al., 2023[165]; WHO, 2023[215]). Pathogenesis of this disease varies depending on the infecting species, and clinical presentations are commonly divided into three categories: Cutaneous (CL), mucocutaneous (MCL), and visceral (VL), the latter being the most dangerous and lethal in 95 % of the cases if untreated, affecting an estimated 50,000 to 90,000 people yearly. In 2022, eight countries - Afghanistan, Algeria, Brazil, Colombia, Iran, Iraq, Peru, and Syria - accounted for 85 % of the new CL cases. Additionally, 21 countries reported deaths due to VL, with Brazil presenting the highest case fatality rate (9.8 %) (Burza et al., 2018[28]; Ruiz-Postigo et al., 2023[165]; WHO, 2023[215]). WHO flagged visceral leishmaniasis for elimination as a public health problem, for which critical actions include early detection, access to medicines, and the development of user-friendly treatments (WHO, 2020[214]). Available medication options include pentavalent antimonials (sodium stibogluconate and meglumine antimoniate), amphotericin B (preferably liposomal), miltefosine, and paromomycin. While efficient for immunocompetent patients, with cure rates over 90 % in six months, there are significant drawbacks. Notably, these drugs are associated with substantial adverse effects and a narrow therapeutic window stemming from their toxicity. They also require trained personnel for administration due to their injectable nature, except for the only oral option, miltefosine (Burza et al., 2018[28]).

The lack of alternative, less toxic, and easier-to-administer pharmacological treatments for leishmaniasis highlights the importance of cheaper and faster pipelines for drug discovery, in which computational methods can be incorporated. CADD (Computer-Aided Drug Design) can be divided into two main categories: Structure-Based (SBDD) or Ligand-Based Drug Design (LBDD). Also, computational analysis of omics-based data may be integrated into the initial steps of an *in silico* pipeline (Yu and MacKerell, 2017[225]; Paananen and Fortino, 2020[137]). Common SBDD methods include molecular docking, molecular dynamics, and the development of structure-based pharmacophore models, and each of these approaches requires a three-dimensional target structure (Shaker et al., 2021[182]). This information can be acquired experimentally, using X-ray diffraction, cryoelectronic microscopy, and nuclear magnetic resonance, or by computational prediction, including homology modeling or *ab initio*, which encompasses artificial intelligence (deep learning) like AlphaFold 2.0, with a backbone accuracy of 0.96 Å RMSD (Jumper et al., 2021[84]; Varadi et al., 2022[205]). The type of obtained data varies based on software, and powerful techniques such as molecular dynamics (MD) provide intricate information about ligand-induced changes to the target or overall stability of the complex at the expense of higher computational cost. Other strategies aim to leverage large databases containing diverse compounds (e.g., PubChem and ChEMBL) or omics data such as STRING (Szklarczyk et al., 2015[194]), which can propose thousands of potential ligand-target or target-target interactions. In addition, constructing target networks may facilitate the exploration of new molecular targets (Rognan, 2017[162]). When structural data is unavailable, or the target is unidentified, researchers can utilize an LBDD approach. These methods are constructed to discriminate between active and inactive ligands through their chemical structure or features. Other types of computer-calculated parameters may guide drug development, for instance, pharmacokinetic and toxicity (ADMET) prediction, Lipinski's rules, and pharmacokinetic modeling, which may be useful for selecting the most promising candidates in terms of safety and route of administration (Yu and MacKerell, 2017[225]; Jumper et al., 2021[84]; Shaker et al., 2021[182]; Wang and Ouyang, 2022[211]). In this perspective, computational methods may play a crucial role in drug repurposing against leishmaniasis and other NTDs, aggregating the cost-effectiveness of *in silico* with an up to 10-fold reduction in cost present in drug repurposing (Nosengo, 2016[133]; Shaker et al., 2021[182]).

As these techniques continue to be employed to propose repurposable candidates against leishmaniasis, it is necessary to systematically assess the information presented in published scientific literature about this context. Considering the heterogeneity of these studies, a scoping review is an adequate approach. This work aimed to summarize *in silico* studies that involved the pursuit of repurposable drugs against leishmaniasis, focusing on the following research question: "What are the main methodologies, results, and outcomes presented on *in silico* studies of antileishmanial drug repurposing?". The extraction involved (i) *in silico* methodology (e.g., software, algorithms, parameters, procedure, databases), (ii) data obtained from these methods (ligands, binding energy, interactions, predicted activity), and outcomes presented (number of candidates, *in vitro* confirmation). Additionally, we identified the most used combinations of methods, frequent and important targets, and critically discussed pharmacological classes that presented promising activity in the literature.

## Methods

### Protocol and registration

This scoping review was conducted based on Joanna Briggs Institute (JBI) methodology (Peters et al., 2020[144]) for scoping reviews and reported according to the PRISMA-ScR checklist (Tricco et al., 2018[200]). The study protocol was previously registered on OSF under the identifier 10.17605/OSF.IO/ K7BT4. Two authors independently performed steps of study selection and data extraction, and a third was consulted as a referee in case of discrepancies.

### Search strategy

The developed search strategy intended to find published studies related to the proposed scope. Relevant keywords/subject headings were selected through a pilot screening of pertinent literature on CADD and leishmaniasis, with iterative refinement of the search queries based on titles, abstracts, and full-text reading to include new terms. Additionally, PubMed results were uploaded to SR-Accelerator (Clark et al., 2020[40]) in the WordFreq tool to detect potential missing keywords. The final search strategy was adapted for three databases (PubMed, Scopus, and Web of Science) to recover studies available up to October 19th, 2023 (updated on April 1st, 2024). Final queries for each database are provided in Supplementary information, Supplement 1.

### Study selection

**Figure 2:**
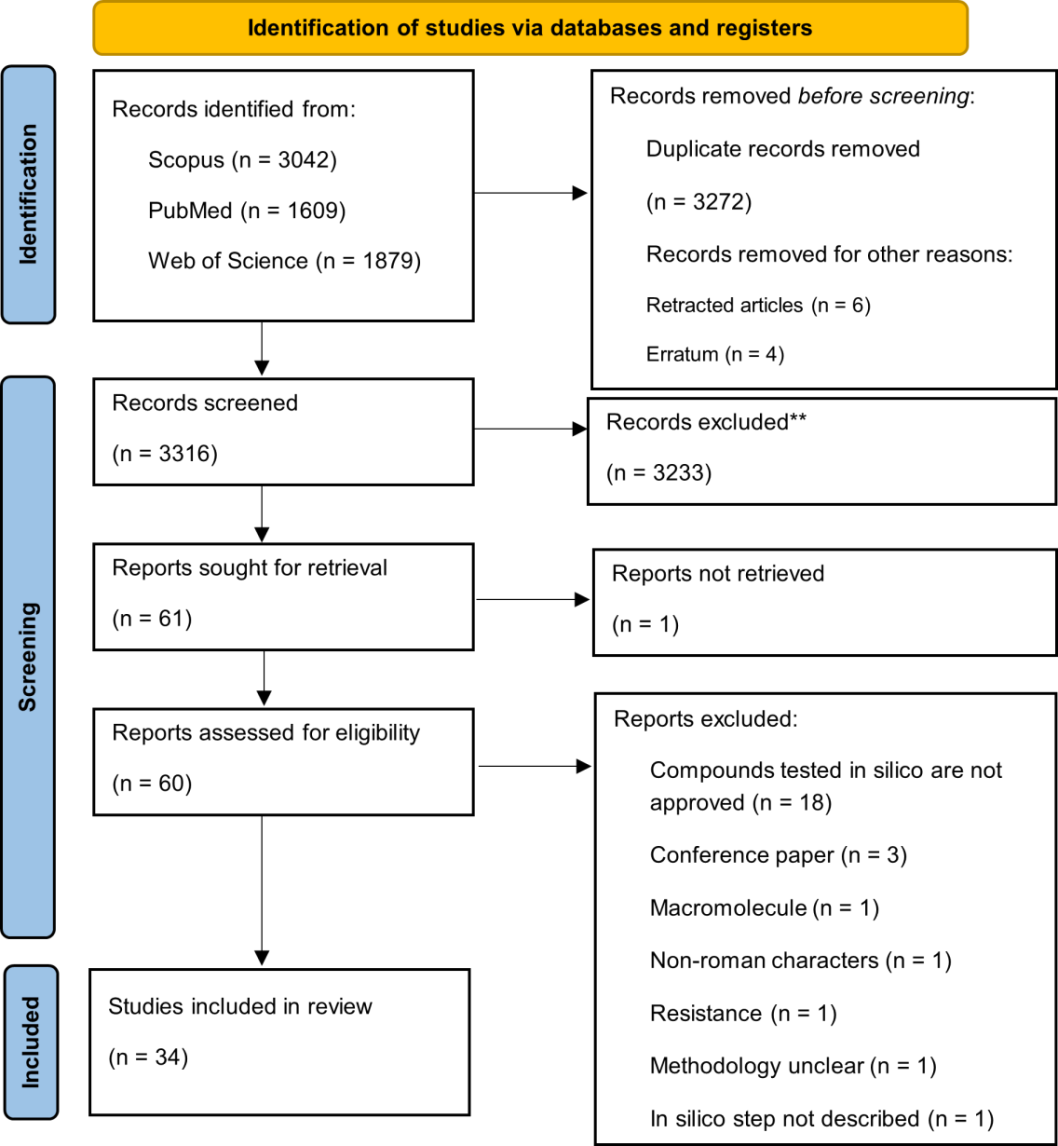
PRISMA flowchart (Page et al., 2021) representing the study selection and inclusion process

Search results were downloaded from each database and duplicate removal was carried out on SR-Accelerator (Clark et al., 2020[40]) Deduplicator tool. Results were uploaded to the Rayyan web app for systematic reviews (Ouzzani et al., 2016[136]) for additional duplicate detection, screening, and labeling by two independent reviewers following the eligibility criteria. Selected studies underwent independent full-text readings by the reviewers for inclusion confirmation. Reasons for further exclusions were registered. Consensus meetings and an additional reviewer, as necessary, solved discrepancies in the decisions. The inclusion process was documented using an adapted PRISMA flow diagram (Page et al., 2021[139]) in Figure 2(Fig. 2).

### Eligibility criteria

This review considered the following criteria to determine article eligibility: (i) primary studies involving the use of any computational method for drug design (CADD) or drug-target identification, (ii) a priori or a posteriori of *in vitro* assays, (iii) aiming at identifying antileishmanial small molecules (iv) through repurposing of approved drugs (Phase I minimum) in at least one country. No publication date restrictions were used. This study did not include reviews, articles written in non-Roman characters, and conference proceedings/papers.

### Data extraction and analysis

The two reviewers extracted data from the included studies with a developed data extraction guide and a corresponding formulary (Supplementary information, Supplement 2), adapted from the JBI Data Extraction Instrument to better represent the type of data presented in these studies (Peters et al., 2020[144]). Extracted data was categorized into seven main groups: General details (author, year, country, and methods used), main objectives, *in silico* methodology, key findings (*in silico* and *in vitro*), drugs, targets, and main outcomes. Data analysis and Chord and Sankey plot construction were conducted using Microsoft® Excel and custom Python scripts (OpenChord and Plotly libraries). In order to prepare a scatter plot summarizing compounds similarity, Ligands Simplified Molecular-Input Line-Entry System (SMILES) codes were retrieved from PubChem (Kim et al., 2023[90]), using a Python script to query the database through the URL-based Application Programming Interface (API). Drug names were defined as the query input, and their respective Canonical SMILES (output) were compiled into a table. The structural similarity was calculated on DataWarrior v6.0.1 (Sander et al., 2015[171]), calculating substructure fragment dictionary-based binary fingerprint descriptors (FragFp) from SMILES. Drugs with a similarity index of over 80 % were considered neighbors, and their combinations were defined as clusters. 

## Results and Discussion

### Study inclusion and characteristics

The removal of duplicates yielded 3316 articles, followed by the removal of 3238 studies based on title and abstract screening. Sixty articles were subjected to full-text appraisal, resulting in 34 studies that met the stipulated eligibility criteria (Figure 2(Fig. 2); Reference in Figure 2: Page et al., 2021[139]). Most excluded studies in full-text reading involved the repurposing of experimental compounds (i.e., not approved) that were not originally synthesized as antileishmanials. Individual exclusion reasons were registered per article (Supplementary information, Supplement 3).

Included studies were from India (n=16; 47 %) (Waugh et al., 2014[213]; Abhishek et al., 2019[1]; Madugula et al., 2021[106]; Tabrez et al., 2021[195][196]; Prava and Pan, 2022[151]; Rai et al., 2022[152]; Kashif and Subbarao, 2023[86]; Prakash and Rai, 2023[149]; Ranjan and Dubey, 2023[154]; Saha et al., 2023[168]; Sarma et al., 2024[174]; Sheikh et al., 2023[187][188]; Nath et al., 2024[129]; Vemula et al., 2024[207]), Brazil (n=3; 9 %) (Silva et al., 2015[190]; Borba et al., 2019[27]; Dos Santos Vasconcelos and Rezende, 2021[50]), Iran (n=3; 9 %) (Shokri et al., 2018[189]; Adinehbeigi et al., 2020[3]; Amiri-Dashatan et al., 2021[6]), USA (n=3; 9 %) (Chavali et al., 2012[36]; Sateriale et al., 2014[175]; Gupta et al., 2022[70]), Mexico (n=2; 6 %) (Nava-Zuazo et al., 2014[130]; Juarez-Saldivar et al., 2023[83]), and Spain (n=2; 6 %) (Santamaría-Aguirre et al., 2023[172]; López-Arencibia et al., 2024[103]). Colombia (Bustamante et al., 2019[29]), Pakistan (Rashid et al., 2024[155]), Thailand (Aiebchun et al., 2023[4]), Tunisia (Harigua-Souiai et al., 2022[73]), and Saudi Arabia (Rub et al., 2019[164]) published one paper each. An overview of included studies is provided in Table 1(Tab. 1) (References in Table 1: Abhishek et al., 2019[1]; Adinehbeigi et al., 2020[3]; Aiebchun et al., 2023;[4] Amiri-Dashatan et al., 2021[6]; Borba et al., 2019[27]; Bustamante et al., 2019[29]; Chavali et al., 2012[36]; Dos Santos Vasconcelos and Rezende, 2021[50]; Gupta et al., 2022[70]; Harigua-Souiai et al., 2022[73]; Juarez-Saldivar et al., 2023[83]; Kashif and Subbarao, 2023[86]; López-Arencibia et al., 2024[103]; Madugula et al., 2021[106]; Nath et al., 2024[129]; Nava-Zuazo et al., 2014[130]; Prakash and Rai, 2023[149]; Prava and Pan, 2022[151]; Rai et al., 2022[152]; Ranjan and Dubey, 2023[154]; Rashid et al., 2024[155]; Rub et al., 2019[164]; Saha et al., 2023[168]; Santamaría-Aguirre et al., 2023[172]; Sarma et al., 2024[174]; Sateriale et al., 2014[175]; Sheikh et al., 2023[187][188]; Shokri et al., 2018[189]; Silva et al., 2015[190]; Tabrez et al., 2021[195][196]; Vemula et al., 2024[207]; Waugh et al., 2014[213]), containing *in silico* methodological main points, targets (and associated species), identified ligands, and a summarized outcome. Raw data and analyses are available for consultation in Supplementary data, Supplement 4.

The most common *in silico* methodology employed was molecular docking (n=25; 73.5 %), followed by MD (n=14; 41 %) and virtual screening (VS) (n=17; 50 %), indicating a prevalence of SBDD. Homology modeling and free energy calculation (PBSA or GBSA) were employed in 14 (41 %) and 10 (29 %) of the studies, respectively. Ten studies utilized omics (29 %), 7 used ADMET/Drug likeness prediction (21 %), and 5 used protein networks or Quantitative Structure-Activity Relationship (QSAR) (15 %). Other methods were used in 4 or less articles. There was an increase in the publishing of articles after 2019, which can be attributed to the more frequent usage of SBDD methods, for instance, docking, MD, and free energy calculation. Between 2012 and 2015, 4 articles utilized omics and metabolic networks. 

**Figure 3:**
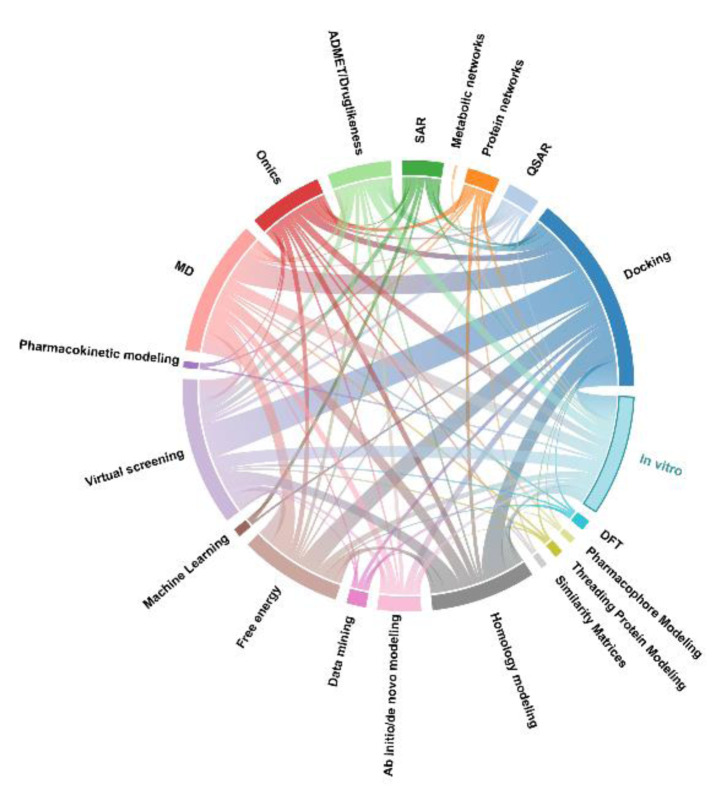
Chord diagram visually representing co-occurrence of *in silico* methods and *in vitro* assays for repurposing against leishmaniasis. The external arc width indicates the proportion of articles that employed each method (e.g. Docking was the most used method, n=25). Each ribbon represents articles that used a combination of the two interconnected methods, and its width corresponds to the number of articles (e.g., the combination of docking and virtual screening was the most frequent, n=17). Colors are categorical for each method.

Regarding method combinations, docking was commonly used in combination with MD (n=14), virtual screening (n=17), homology modeling (n=17), and free energy calculation (n=10). Omics techniques were frequently used with docking (n=5), protein networks (n=4), and homology modeling (n=2). AutoDock Vina was the most used software for molecular docking (n=13), followed by Glide (Schrodinger) (n=7), and GOLD (n=3). For MD, GROMACS, a free software, was used in 7 studies, followed by Desmond (Schrodinger) (n=6), and NAMD2 (n=1). For free energy calculation based on molecular mechanics (MM), GBSA was employed in 6 articles and PBSA in 3 articles (Supplementary Data, Supplement 4). A visual summary of the number of articles containing each pairwise combination of methods is presented as a chord diagram in Figure 3(Fig. 3). A chord diagram can be used to visualize the relationships between data, which is encoded in the form of a circle, with arcs for each category of data, linked by ribbons of proportional width to the weight of the connection between categories.

### Docking, molecular dynamics, and Poisson-Boltzmann/Generalized Born Surface Area

Molecular docking and molecular dynamics (MD) are widely employed methods of SBDD, focused on modeling ligand binding to specific targets, often proteins. Docking involves two main components: a sampling algorithm for generating binding conformations and a scoring function to rank them. Different mathematical approaches can be used for modeling these steps, including the combination of multiple algorithms hierarchically. Ligands are often treated as flexible entities with up to six degrees of freedom (without altering the bond length and angle), and the protein is a rigid or semi-rigid body (Friesner et al., 2004[62]; Meng et al., 2011[112]). Docking-based virtual screening is valuable for enriching candidates from compound libraries for further testing, increasing the number of hits (Śledź and Caflisch, 2018[191]). As a tradeoff for speed, it relies upon several simplifications. Results are not completely translatable to biological activity, and overall performance highly depends on the software and system being studied (Wang et al., 2016[212]). Unlike docking simulations, MD simulations provide an additional layer of complexity by considering the behavior of all atoms within the system, including ions and solvents. At its core, MD focuses on solving Newtonian equations of motion, iteratively determining the forces over each atom, allowing for predicting their trajectory over a specified time (Hollingsworth and Dror, 2018[75]).

Free energy calculation offers valuable insight into complex formation favorability. Alchemical Free Energy (AFE) methods are very accurate but require calculating intermediaries between two physical states, such as the binding-unbinding of a ligand or the mutation of a residue, requiring extensive computational resources and are impractical for screening several candidates (Wang et al., 2019[210]; Mey et al., 2020[113]). PBSA and GBSA methods, based on molecular mechanics (MM) end-point calculations, reduce computation time by considering only final states. These two approaches differ in the computation of polar contribution between solute and solvent: For PBSA, the Poisson-Boltzmann equation is used, while GBSA provides a faster strategy of approximation based on the generalized Born model (Mongan et al., 2007[117]). Binding energy can be estimated by averaging snapshots from MD trajectories or a single minimized structure, with both approaches yielding similar accuracy. Using a single structure is advantageous for rescoring docking results with PB/GBSA calculations, enhancing pose determination, and differentiating inhibitors from non-inhibitors (Kuhn et al., 2005[95]; Sun et al., 2014[193]).

Abhishek et al. (2019[1]) specifically sought to explore the antileishmanial mechanism of action of auranofin, an antirheumatic agent, using docking, MD, and MM/GBSA to evaluate the binding affinity of auranofin and its intermediates: tetraacetylthioglucose (TAG)-gold and triethylphosphine (TP)-gold against *L. infantum* TryR. Previously obtained crystal structures indicate that Au(I) is transferred to TryR, although the exact mechanism is not determined (Ilari et al., 2012[79]). Docking results corroborated the dimeric interface of TryR as the binding site. The drug and intermediates presented good binding stability, conserving intermolecular interactions throughout the MD. A lower binding energy was observed for the complete molecule (-26.62 kcal/mol), indicating that the formation of intermediates and the Cys-Au-Cys adduct in the catalytic site may take place after the complete auranofin molecule binds to TryR. *In silico* methods may also be utilized after *in vitro* experiments to further elucidate ligand-target interactions. For instance, Aiebchun et al. (2023[4]) further explored *in silico* interactions of afatinib and lapatinib with MAP3K, utilizing docking and MD. Their study identified important interactions involving H-bonds with Glu94 and Asp193 for afatinib, Glu94 for lapatinib, and a halogen bond with Glu206. MD simulations, performed in triplicate using GROMACS, showed that afatinib stabilized in the simulation after 80 ns, while lapatinib stabilized earlier, around 30 ns, as analyzed by Root-mean-square deviation (RMSD). Analysis of the stability of a protein-ligand complex in simulation trajectory can be done by plotting the protein RMSD, which measures its structural variation compared to the initial conformation, indicating how much the structure deviates from the starting condition over time. Equilibration, marked by stabilized RMSD values, indicates successful ligand recognition. A limitation of this measurement is that the entire structure is evaluated, and regions of high flexibility may increase the RMSD value even if the binding pocket is stable. Root-mean-square fluctuation (RMSF), based on time average RMSD, determines the backbone fluctuation of residues to identify stable and mobile regions. A third measurement, the radius of gyration, represents time-dependent protein folding (or compactness) (Hernández-Rodríguez et al., 2016[74]). Finally, the last 20 ns from each trajectory were chosen for interaction analysis, confirming a stronger lapatinib interaction with MAPK3 through three H-bonds with Ser74, Glu94, and Met133. In contrast, the MM/PBSA results were similar for both drugs regarding ΔGbind, and van der Waals forces were the main contributors to stabilizing the molecules.

Adinehbeigi et al. (2020[3]) listed 10 promising molecules obtained from a docking-based VS against arabinono-1,4-lactone oxidase from L. donovani (LdALO). The virtual screening was based on a ligand library of 2500 approved drugs from DrugBank database, structurally optimized with the LigPrep module from Schrödinger, and generated up to 32 stereoisomers, totalizing 5000 ligands. Docking was conducted with AutoDock Vina inside PyRx. Considering the binding energy from Vina, the best molecule was suramin (-28.9 kJ/mol), and interactions occurred inside the active site. Similarly, Kashif and Subbarao (2023[86]) ran a virtual screening campaign of 4000 ligands, mostly approved drugs, obtained from the ZINC database against glutamine synthetase from L. major. Additionally, 100 ns MD simulations were performed with the top four molecules to evaluate stability in the binding pocket. Using the stable phase of the simulation, MM/PBSA was calculated to rank promising candidates.

Harigua-Souiai et al. (2022[73]) utilized reverse docking to evaluate 19 approved drugs binding to seven targets. Unlike traditional docking methods which tests multiple ligands against the same target, reverse docking evaluates a single ligand against multiple targets. The drugs were identified through machine learning/SAR without prior knowledge of targets. They compared the binding energies from Vina and established a threshold using the mean value from all results. A secondary criterion was the contact rate, which is the number of close contacts (<3.5 Å) divided by the number of total atoms in the molecule. A ligand-target pair with at least 50 % of the contact rate of the co-crystallized molecule was considered a good fit. Such a normalization step is relevant to critically interpret reverse docking results, as scoring functions are biased towards proteins with large or hydrophobic binding cavities (Luo et al., 2017[104]). Four potential pairs were identified: ganciclovir/PTR1, domperidone/TryR, prilocaine/MAPK10 and albendazole/NMT. Gupta et al. (2022[70]) followed a similar strategy after determining posaconazole and lansoprazole antileishmanial effect in vitro, using data mining to list possible targets and verifying their affinity through induced-fit docking in an implicit membrane, MM/GBSA rescoring, and 100 ns MD simulations inside the Schrodinger suite. The induced-fit docking considers ligand-induced alterations in protein conformation, more accurately representing the binding event than fully rigid protein docking (Miller et al., 2021[114]). Lansoprazole displayed a stable interaction with the ATP-binding site of a calcium motive P-type ATPase, stabilized mainly by h-bonds. Posaconazole presented an excellent affinity for its putative target, sterol 14-alpha demethylase (ligand RMSD < 2.0 Å after 10 ns). Finally, Rashid et al. (2024[155]) further evaluated ligands obtained from a 3D-QSAR model through docking and determined the binding affinity of nystatin against three structures of L. mexicana arginase.

Some studies presented small methodological variations in the MD simulations. Ranjan and Dubey (2023[154]), Saha et al. (2023[168]), Sheikh et al. (2023[187][188]), and Vemula et al. (2024[207]) performed MD trajectories of 100 ns. The longest one was provided by Nath et al. (2024[129]), which simulated L. donovani mitochondrial DNA primase for 300 ns on Desmond. This work also took an innovative approach to visualize correlations in residue fluctuations. Applying Principal Component Analysis (PCA) to compute eigenvalues and plotting a Dynamical Cross-Correlation Matrix (DCCM) demonstrated primase bound to benfotiamine and capecitabine significantly differed from the apo state. In contrast, Adinehbeigi et al. (2020[3]) performed a very short MD simulation of only 10 ns to optimize a homology model structure. A critical aspect of MD is the proper choice of force field, which are equations and parameters that represent the atomic potential energy and significantly influence the validity of the simulation (Hollingsworth and Dror, 2018[75]). Mainly, studies utilized the commercial OPLS3 (and 3e), GROMOS96, and OPLS_2005 force fields, and for solvent models, TIP3P and SPC. OPLS3e is newer and has superior accuracy among OPLS force fields, with around 20 % less overall RMS error than OPLS3, which is 30 % more accurate than OLPS_2005. (Roos et al., 2019[163]). In a comparative study, AMBER and CHARMM force fields better represented the side-chains rotamer population than OPLS and GROMOCS (Petrović et al., 2018[145]). TIP3P and SPC are three-point charge rigid water models still widely employed for computational efficiency. Even so, four-point charge models like TIP4P account for water dissociation events, which may influence certain protein systems (Emperador et al., 2021[53]). In most simulations, the temperature was set to 300K, and only one study carried out the simulation at 310K. Although the impact of temperature variation in MD of ligand-protein complexes is not fully established, it is known that proteins can be sensitive to thermal variations. The receptor binding motif of SARS-CoV-2 spike protein adopts an alternative closed conformation around 40ºC in MD, rendering it less suitable for binding to human ACE2 (Rath and Kumar, 2020[156]).

A strong trend towards SBDD, particularly molecular docking, is evident in recent research on drug repurposing against leishmaniasis, yielding 96 repurposing candidates. The methodologies varied essentially in terms of software, specific parameters, and number of ligands screened. Also, ZINC and DrugBank were the preferred libraries to download approved compounds. Among all works, the largest screening was done by Sheikh et al. (2023[187]), with 8630 drugs from ZINC. In another article, authors utilized a similar library from DrugBank as well, with 8500 drugs (Sheikh et al., 2023[188]). This dataset likely presents redundancies, considering that authors reported glimepiride and Amaryl (trade name) as separate chemical entities. Currently, there are around 2700 approved small molecules in DrugBank (https://go.drugbank.com/stats). Considering articles that employed virtual screening through Vina, none applied normalization measures to deal with a known bias towards high MW compounds, such as the number of heavy atoms, which may accumulate large molecules in top positions that can be false positives. (Carta et al., 2007[32]; Xu et al., 2022[221]). In contrast, Glide is less prone to overestimate affinity from molecular size (Boittier et al., 2020[25]). Additionally, most methodologies included careful preparation of proteins and ligands, such as correcting ionization states in pH 7.0, energy minimization of the receptor, and tautomer generation, which increases results reliability (Adinehbeigi et al., 2020[3]; Gupta et al., 2022[70]; Rai et al., 2022[152]; Aiebchun et al., 2023[4]; Ranjan and Dubey, 2023[154]; Saha et al., 2023[168]; Santamaría-Aguirre et al., 2023[172]; Sheikh et al., 2023[187]; Vemula et al., 2024[207]).

### Protein structure prediction

To perform virtual screening, Adinehbeigi et al. (2020[3]) identified the necessity to use ab initio and threading models to generate a protein model, due to the unavailability of an experimentally determined structure with satisfying homology. Besides selecting experimentally obtained structures from PDB or other databases, which often limits the investigation of novel targets, authors may utilize structure prediction by homology modeling, ab initio modeling, or threading methods. In contrast to experimental structures, more protein sequences are available in repositories such as NCBI. An aminoacidic sequence is sufficient to choose a computational method for prediction, which will depend on further information available (Jumper et al., 2021[84]). 

Homology (or comparative) modeling involves selecting an experimentally determined structure (a template) with enough sequence similarity to the desired target (the model). More than 25 % of similarity indicates that both proteins should adopt a similar tridimensional conformation, but other factors, such as phylogenetic proximity, also play a role (Muhammed and Aki-Yalcin, 2019[120]). Threading modeling differs from homology modeling in the alignment, which is done considering the tertiary structure of the template instead of simply sequence-sequence alignment. This strategy allows the utilization of templates with lesser similarity, but similar folding. In the same context, ab initio modeling aims to generate the protein structure by energy minimization or folding simulation. The main difficulty of ab initio is the higher computational requirements for simulation and the development of reliable mathematical models to represent the protein folding and obtain the lowest energy conformation (Huang et al., 2023[76]).

The authors chose to generate multiple models, using Robetta, I-TASSER, MUSTER, and LOMETS, employing both ab initio and threading methods, and further selected and confirmed the best model based on quality evaluation and validation with three web servers: Qualitative model energy analysis (QMEAN), RAMPAGE and PROCHECK. QMEAN provides a scoring function based on a linear combination of structural descriptors, including long-range interactions of beta-carbons and all-atom types, torsion angle potential of consecutive residues, solvation potential of residues, and two descriptors for the agreement of predicted and calculated secondary structure and solvent accessibility (Benkert et al., 2011[21]). RAMPAGE and PROCHECK are tools capable of generating a Ramachandran plot, which is useful for determining the overall quality of the structure in terms of residues in allowed regions. The z-score from QMEAN was used to select the best model between all servers. The model from Robetta was selected for further comparison based on a Ramachandran plot from PROCHECK, which validated the z-score result. Posterior structure refinement was performed with the 3d refine server and a short MD simulation of 10 ns using NAMD2.

### (Q)SAR models

Ligand-based techniques are useful when data regarding active/inactive ligands or a collection of lead compounds against a particular organism exists. A fundamental example involves conducting a similarity search from a ligand, assuming that compounds with similar structures are more likely to elicit an effect on the same organism or target. A common measure of similarity is the Tanimoto coefficient. Structure-activity relationship (SAR) is another option, which can be categorical or quantitative (QSAR). SAR generally involves generating descripttors to numerically represent the molecules and applying an algorithm to determine a relationship between descriptors and bioactivity (Yu and MacKerell, 2017[225]). Several databases containing results of biological assays against targets or whole cells may provide the required data to construct the model, such as PubChem (Kim et al., 2023[90]), DrugBank (Knox et al., 2011[92]), ChEMBL (Gaulton et al., 2012[65]) and BindingDB (Liu et al., 2007[102]). 

Molecular descriptors may represent physicochemical parameters, quantum properties, molecular connectivity, or topology, which can be experimentally defined or calculated by different methods (called theoretical descriptors). Software for descriptor calculation includes Mordred, PaDEL-Descriptor, BlueDesc, ChemoPy, and PyDPI (Moriwaki et al., 2018[119]). These descriptors can be codified as fingerprints (binary code), in which the presence of a characteristic or substructure is represented by “1”. The correlation of these variables can be established through statistical methodologies such as chemometric or machine learning algorithms (Cheng et al., 2013[38]; Sahoo et al., 2016[169]). The input may vary in dimensionality, starting from 1D-QSAR, which utilizes general molecular properties like LogP. Additionally, 2D and 3D-QSAR consider atom bonding and interaction fields in two or three dimensions. 3D-QSAR may take advantage of experimentally determined receptor structures, so molecular alignment will consider the biologically important conformations. The tridimensional geometry can be optimized through molecular mechanics, semi-empirical, or quantum methods, increasing complexity. Quantum methods are computationally heavy but provide high accuracy for considering the molecular electron distribution. At the same time, semi-empirical approaches are based on approximations of quantum methods to speed up the calculation (Verma et al., 2010[209]).

Harigua-Souiai et al. (2022[73]) constructed four SAR models using information deposited in PubChem, related to two bioassays against *Leishmania *promastigotes. The initial dataset contained 65,057 molecules. Subsequent reduction of the dataset involved performing ROS, RUS, and two sub-samplings to 10 % and 1 %, resulting in five different datasets. The descriptors were generated using the RDkit package in Python as five binary molecular fingerprints. Sci-kit learn was used to develop the machine-learning model, and four algorithms were tested: Linear regression, gradient boosting, random forest, and support vector machine. Algorithm performance was determined by sensitivity, specificity, precision, balanced accuracy, and F1-score, resulting in SVM and RF being the most accurate (0.72 balanced accuracy). The original and ROS datasets produced comparable accuracy. The F1-score, which considers sensitivity and precision, was 0.37 (RF) and 0.39 (SVM). The authors associated the high number of false positives because of an imbalanced dataset, as most molecules were inactive. This model was employed for SAR-based virtual screening of 1065 FDA-approved drugs, resulting in 19 molecules (Table 1(Tab. 1)), of which 7 had previous reports of antileishmanial activity.

An advantage of (Q)SAR compared to SBDD methods lies in a faster screening process, allowing it to handle larger amounts of data. Madugula et al. (2021[106]), aiming to find repurposable drugs against multiple diseases, employed a SAR model available online, PASS. This tool can classify compounds ranging from 50 to 1250 Daltons into more than 4000 categories of biological activity with over 95 % of accuracy (Filimonov et al., 2014[58]). The authors processed 1671 approved drugs using 1444 2D molecular descriptors and machine learning algorithms, PCA, and k-means, further grouping the molecules into 9 clusters. After determining molecules with a probability of activity (Pa ≥ 0.5) using PASS, 4 drugs were deemed as potentially antileishmanial, including artemether and artemisinin. The purpose of clustering was to provide additional insight. By structural similarity, members of the same cluster could share biological activities based on their original and predicted indication.

Rashid et al. (2024[155]) built a more complex receptor-based 3D QSAR model in FLARE v5, accounting for protein-ligand interactions. This model was focused on 50 natural compounds from Calotropis procera with antibacterial and antiprotozoal activity. 3D descriptors were calculated using the XED force field and considered the antimicrobial peptide LL-37 (PDB: ID 5NNM) as a receptor. Furthermore, important features of the molecules were mapped, regarding favorable hydrophobic and polar regions for antimicrobial activity. A virtual screening was carried out with the model, and the top 10 compounds with pIC_50_ < 4.8 were considered promising based on their similarity with the chemical space of the training set. The results indicated nystatin has antileishmanial potential. Accordingly, preclinical studies show nystatin cream can reduce *L. amazonensis* infection, considering a cutaneous leishmaniasis animal model with BALB/C mice (Gonçalves-Oliveira et al., 2023[68]).

### Pharmacokinetics, toxicity, and drug-likeness

ADMET prediction is a relevant *in silico* technique for drug discovery, particularly for novel compounds and lead optimization. Current models, such as Boiled-EGG to estimate intestinal absorption and brain-blood barrier crossing (Daina and Zoete, 2016[46]), are freely available online. Similarly to QSAR, ADMET models are based on the correlation between compound descriptors and pharmacokinetic/toxicity parameters experimentally determined, namely QSPR. Likewise, data inference may be done by machine learning algorithms or statistical methods, such as SVM, ANN, PCA, and PLS. Structurally, some chemical groups are also notably associated with toxic effects, so-called toxophores (Cheng et al., 2013[38]; Chen et al., 2024[37]). According to the estimated parameter, the output value may be categorical, numeric, or a probability range. Common outputs include physicochemical properties, Caco-2 cell permeability, plasmatic protein binding, interaction with CYP450 enzymes, carcinogenicity, and genotoxicity. Apart from lead-compounds pre-evaluation, ADMET may be used for virtual screening, to prioritize favorable compounds from a large library (Rognan, 2017[162]). Drug-likeness, on the other hand, generally consists in rule-based filters that are statistically likely to be presented by drugs, which can be certain functional groups or physicochemical properties. Lipinski's rule of five is one of the most well-established filters. It consists in selecting molecules with (i) MW < 500 Da, (ii) LogP < 5, (iii) less than 5 h-bond donors, (iv) less than ten h-bond acceptors, and (v) molar refractivity in the range of 40-130. Other medicinal chemists proposed their own set of rules, such as Veber, Ghose, and Egan (Ursu et al., 2011[201]; Kralj et al., 2023[93]).

Nava-Zuazo et al. (2014[130]) evaluated the toxicity of nitazoxanide using ACD/ ToxSuite, to determine murine acute toxicity on both intraperitoneal and oral routes, as well as the inhibition of CYP3A4/2D6/1A2 an the effect on the hERG channel. Results were in accordance with the high tolerability of nitazoxanide (Murphy and Friedmann, 1985[126]), presenting high LD_50_ and low probability of inhibition of CYP450 isoforms and hERG. For other works, the evaluation of bortezomib also indicated no inhibition of five CYP450 isoforms (López-Arencibia et al., 2024[103]). Notably, bazedoxifene (AMES test), glimepiride, and imatinib (hepatic) presented toxicity alerts (Ranjan and Dubey, 2023[154]). Differently, Vemula et al. (2024[207]) used ProTox-II to establish toxicity classes for cabergoline (class 3), raloxifene (class 4), and formoterol (class 5) based on their predicted LD_50_ dose (200, 400, and 3130 mg/kg, respectively).

The authors also applied the filter-based rules of Lipinski, allowing a maximum of 1 violation. Nath et al. (2024[129]) used it to prioritize bioavailable drugs obtained from docking for MD simulations and identified no violations. Additionally, they evaluated ADMET profiles in pkCSM, for which all candidates presented good predicted properties. Following the same methodology, Ranjan and Dubey (2023[154]) detected one violation for abemaciclib, and glimepiride (Amaryl) showed less Caco-2 permeability than other drugs in pkCSM. Similarly, Prakash and Rai (2023[149]) evaluated retinoic acid and its derivatives and verified that retinoic acid and adapalene had zero and one violation, respectively. López-Arencibia et al. (2024[103]), extensively employed filter-based exclusion in their investigation of COVID-box compounds, using five sets of rules from Lipinski, Ghose, Veber, Egan, and Muegge. Results indicated that ABT239, a drug investigated for cognitive disorders, did not violate any rules, and bortezomib only violated one Veber's rule. In comparison, miltefosine presented violations in four rules: two in Ghose's and Muegge's, and one in Veber's and Egans's.

An important aspect of repurposing approved drugs is the preexisting knowledge of their pharmacokinetic profile acquired from clinical trials or other studies with humans. Bustamante et al. (2019[29]) modeled different dosages for rifabutin and perphenazine, considering parameters available in the literature. This approach revealed that the oral administration of these drugs would be insufficient to reach a leishmanicidal plasmatic concentration, considering the EC_50_ determined *in vitro*. This methodology could be applied to other works, providing additional criteria for ranking promising candidates for *in vivo* assessment. Further insight could be provided by physiologically based pharmacokinetic models (PBPK), which incorporate the compartmental aspect of distribution in the different tissues (Jones and Rowland-Yeo, 2013[82]).

### Density functional theory

Only Sheikh et al. (2023[187]) applied density functional theory (DFT) to stipulate drug reactivity. This technique is based on quantum theory and used in drug design for calculating the electronic structures of molecules. Notably, DFT is concerned with calculating the kinetic energy of electrons and electron-electron interaction energy (Mardirossian and Head-Gordon, 2017[109]). The authors used the Jaguar module from Schrodinger, which approaches DFT through a pseudospectral (PS) method, aiming to increase efficiency for larger biological systems (Bochevarov et al., 2013[24]). An important aspect of DFT is the so-called “basis set”, which are functions used to construct the desired molecular orbitals. The authors utilized the B3LYP-D-D3 functional at the 6-31*G basis set, which balances accuracy and low computational cost. Important global molecular properties can be extrapolated from the calculated HOMO-LUMO difference: Electronegativity, electrophilicity, hardness, and softness. The gap (difference) between the energies of HOMO and LUMO is inversely proportional to molecular stability. Another technique based on DFT, Molecular Electrostatic Potential (MEP), allows the mapping of electronic distribution. Coupled with classical MM-SBDD methods like MD, DFT may provide further insight into molecular interactions and covalent bonding. (Ye et al., 2022[222]). 

A comparison between the two proposed antileishmanials, ceftaroline fosamil and rimegepant, and a current drug, pentaminidine, was carried out. Rimegepant and pentamidine presented similar reactivities, considering their E_gap_ (5.09 eV and 5.13 eV, respectively), and ceftaroline fosamil was the most reactive molecule (0.50 eV). The MEP results seem to align with predicted h-bonding, with most the most electrophilic regions acting as h-bond donors to the protein residues.

### Genomics and proteomics

Leveraging the available omics data on *Leishmania *presents a promising approach for discovering novel targets for drug repurposing. Different experimental high-throughput methods, such as genome sequencing, genome-wide association, and mass spectrometry, are available for genomic and proteomic data acquisition. While genomics is concerned with obtaining genetic information about a cell, tissue, or organism and associating this information with a phenotype, commonly through single nucleotide polymorphisms (SNPs) proteomics is focused on detecting or quantifying the current-state protein content of the biological sample (Paananen and Fortino, 2020[137]). This data can be processed using bioinformatics, including functional prediction, gene ontology, phylogenetic tree assembly, and protein-protein interaction network analysis. Fundamentally, genomic and proteomic data allows target prioritization based on essentiality to the pathogen, non-homology to humans, specificity to pathogenic counterparts of a genus, and the potential to be modulated by a drug-like compound (druggability) (Xia, 2017[220]; Serral et al., 2021[181]). BLAST is a popular algorithm for comparing genetic sequences. An alternative version, named BLASTp, is used for protein sequences.

Prava and Pan (2022[151]) investigated the proteomes of 11 *Leishmania *species to identify potential targets. By focusing on proteins absent in the human proteome and similar between all species, they arrived at 3605 “core proteins” and predicted their functions and subcellular location using Gene Ontology (GO) via Blast2GO. Gene Ontology is rooted in the principle of conserved genes also preserving similar functions and localization throughout eukaryotes (Ashburner et al., 2000[10]). Likewise, Abhishek et al. (2019[1]) utilized ClustalW to determine the conservation of trypanothione reductase and analogous enzymes between different parasites, including *Leishmania, Trypanosoma and Plasmodium* genus. The residues involved in auranofin binding exhibited high conservation among the evaluated species, particularly the two catalytic cysteines, which could explain the cross-species activity observed *in vitro*. Vemula et al. (2024[207]) also utilized BLAST to determine the conversation of trypanothione synthetase among different *Leishmania *species, and a pairwise alignment in Clustal Omega between *L. major* and *L. donovani*, which resulted in 98.47 % similarity. In the work of Nath et al. (2024[129]), they confirmed the dissimilarity of *L. donovani* mitochondrial primase 1 in relation to human proteome through BLASTp.

Borba et al. (2019[27]), seeking to determine protein kinases present in *L. infantum* and *L. braziliensis* proteomes, utilized Kinannote v1.0 to classify them into groups, families and subfamilies. Unclassified kinases were compared to an *L. major* kinome for clarifycation since this kinome was already elucidated. In sequence, a kinase domain prediction was carried out using InterproScan. Similarly to Prava and Pan (2022[151]), they compared the *Leishmania *kinome with *H. sapiens*, to prioritize the proteins unique to the parasite and discard human homologs, via BLASTp. Orthologs (conserved proteins) from the three species were also verified. This steps are relevant, since targeting a conserved protein absent in humans increases the probability of parasite-selective pan-species drug (Arendse et al., 2021[9]). An essentiality measure was also performed by determining orthologues to *T. brucei* kinases with lethal siRNA phenotypes. Finally, 30 prioritized kinases were compared to drug targets deposited in DrugBank and Kinase SARfari to determine repurposing candidates, resulting in 11 targets with high sequence similarity associated with 42 approved drugs.

Bustamante et al. (2019[29]), Sateriale et al. (2014[175]), and Silva et al. (2015[190]) followed similar pipelines, which consisted in comparing sequences from *Leishmania *genome or proteome to known drug targets present in DrugBank. Interestingly, the E-values used as cutoff varied (10-8, 10-100 and 10-5), as well as the number of biomolecules used for comparison, which ranged from the complete proteome of five *Leishmania *species to specific genes annotated to be involved in energy metabolism.

### Protein-protein interaction networks (PPINs)

Since a limited number of validated targets are described in the literature, the construction of protein networks can be a starting point for target prioritization. Mapping protein interactomes experimentally involves complex assays with inherent limitations, such as yeast-2-hybrid and affinity purification in tandem with mass spectrometry (Snider et al., 2015[192]). *In silico* prediction of PPINs requires fewer resources and may facilitate drug discovery by leveraging existing knowledge on PPIs. The prediction can be defined in a binary approach, classifying a protein pair as positive or negative for interaction. More sophisticated analysis can be done by predicting the protein's interaction interface, which may serve as a specific binding site for molecules. Topological analyses, by measuring betweenness centrality and node degree distribution, can be used to determine hubs and bottlenecks of the network: Hubs are proteins involved in a high number of interactions, while bottlenecks serve as intermediates between nodes (Murakami et al., 2017[125]).

The studies employed PPINs in several ways to find repurposing candidates. Amiri-Dashatan et al. (2021[6]) constructed an *L. major* PPIN using the metacyclic stage proteome and the STRING database. Through topological analyses, they pinpointed “essential proteins”, with high values of node degree and betweenness centrality, as potential targets. Among these, pyruvate kinase was selected as a target for subsequent docking-based virtual screening. 

Prava and Pan (2022[151]) utilized PPINs more extensively. They opted to construct a network containing only human non-homologous proteins and conserved throughout 11 species of *Leishmania*. Employing BLASTp algorithm for comparison analysis resulted in 3605 core proteins. Further selection criteria were defined by gene ontology via Blast2GO, which encompass properties such as subcellular localization, molecular function, and biological processes related to these proteins. The construction of the PPIN was carried out using data from the STRING database and topology analysis with Network Analyzer, which resulted in 8 proteins with high node degree and betweenness centrality. Potential drugs can be identified by comparison with databases containing known drug targets (druggability) and by assessing their similarity to proteins within the network. The authors chose DrugBank as the source of ligand-target information and detected three targets with known ligands among the hub proteins. 

Since PPINs may introduce novel targets, their combination with structure prediction techniques is particularly useful for exploring them with SBDD methods. Prava and Pan (2022[151]) predicted the structure of the targets with Robetta to evaluate ligand affinity using docking (on Glide). Two were associated with approved drugs: eIF3 and RPL2 with artenimol and omacetaxine mepesuccinate, respectively.

Borba et al. (2019[27]) took a more specific approach to PPINs, by choosing only the kinomes (set of protein kinases) of *L. infantum* and *L. braziliensis* to build the network. The PPIN was built via STRING, and topological analysis (CytoNCS) was used as a secondary strategy for target prioritization. In contrast, Bustamante et al. (2019[29]) first identified hits from drug targets in DrugBank based on similarity to the proteins of five pathogenic species of *Leishmania *from the TriTrypDB database (hits were present in at least two species), resulting in 33 drugs and 80 targets. Subsequently, the authors also compared these targets with 1273 proteins from a previously constructed in-house PPIN of *L. braziliensis*. They detected three hub proteins among the previous hits, associated with 12 drugs. Function annotation and metabolic pathway information was retrieved from KEGG database.

A more systematic attempt was undertaken by Dos Santos Vasconcelos and Rezende (2021[50]). Firstly, they collected predicted protein sequences from *L. braziliensis* and *L. infantum* from TriTrypDB, and all known drug targets deposited in BindingDB with high affinity (<10 μM). Afterward, tridimensional structures from each target were collected in PDB or the SwissModel repository. Instead of comparing *Leishmania *proteins and drug targets only by homology, a detailed evaluation was conducted by five similarity matrices, comparing drug binding sites, druggability of similar binding sites, subcellular locations, biological processes, and molecular functions. The authors considered the protein promising if the binding site and its druggability were equivalent to the target. Additionally, two of three secondary requisites (Molecular Function, Biological Process, or Subcellular Location) needed to be similar as well. The PPIN of *L. braziliensis* and *L. infantum* was used to filter targets based on their essentiality in the network. To further refine the results, a cutoff of 50 % similarity was used for proteins homologous to the human proteome. A final selection was made to filter only proteins that displayed evidence of expression in the parasite amastigote stage, using gene expression information in the SRA database. A final set of 9 proteins (4 from *L. braziliensis* and 5 from *L. infantum*) fulfilled all requirements, with 145 repurposable compounds associated with BindingDB.

It is worth noting that none of the included studies employing PPINs sought to establish an interactome as a target but relied on topology metrics to prioritize proteins, which could be essential. Although the authors focused on node degree and betweenness to propose targets, these measures do not necessarily indicate a drug target. Drug targets may present lower connectivity than non-targets and instead interact with the most highly connected proteins, which tend to group in modules. Coreness, modularity, and eccentricity could be more suitable topological parameters to determine target-like proteins in a PPIN (Feng et al., 2017[55]).

### Structural similarity of hit compounds

**Figure 4:**
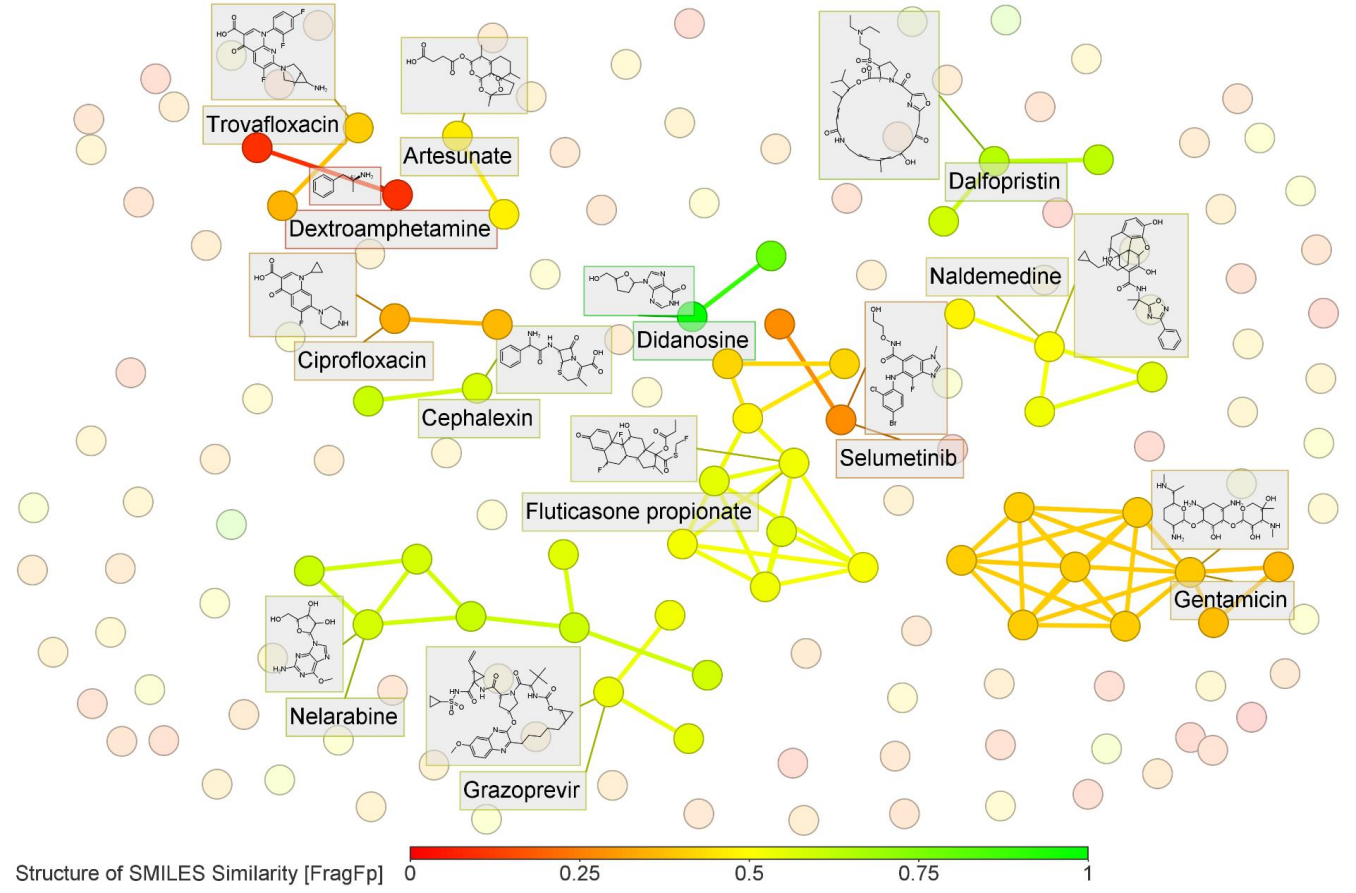
Scatter plot exhibiting molecules clusterization based on structural similarity (FragFp descriptors cutoff for neighbors ≥ 0.80, displayed as links between nodes). Didanosine was selected randomly as a reference for overall similarity calculation. 13 clusters (with 2 or more drugs) were identified. The drug with the highest neighbor counts was selected to represent each cluster (name and structure displayed). Single-drug clusters are represented in faded colors.

A total of 154 unique ligands were identified as potential drugs for repurposing, of which 21 were not associated with a target. The most frequent drug was nilotinib (n=3 studies). Abemaciclib, ciclesonide, dutasteride, imatinib, irinotecan, lidocaine, nitazoxanide, rifabutin, simeprevir, trametinib, and valrubicin appeared in 2 studies, each. For summarization, molecules were grouped considering structural similarity. Upon clusterization based on FragFp descriptors, 13 clusters with ≥ 2 molecules were identified, encompassing 49 drugs (Figure 4(Fig. 4)). The most populated clusters, represented herein by gentamicin and fluticasone propionate, had 9 molecules each, characterized by aminoglycoside antibiotics (gentamicin, paromomycin, neomycin, tobramycin, kanamycin, framycetin), antimalarial (artenimol and artemether), and an antirheumatic (d-glucosamine) for the first cluster and corticosteroids (budesonide, fluticasone propionate, fluticasone furoate, ciclesonide, flunisolide, fluticasone, deflazacort, mometasone, and beclomethasone) for the second cluster. Other clusters were represented by nelarabine (antineoplastic), dalfopristin (streptogramin), dextroamphetamine (sympathomimetic), artesunate (antimalarial), selumetinib (MEK inhibitor), cephalexin (cephalosporin), ciprofloxacin (fluoroquinolone), didanosine (reverse transcriptase inhibitor), grazoprevir (protease inhibitor), naldemedine (mu-receptor antagonist), and trovafloxacin (fluoroquinolone).

### In vitro assays

Eighteen articles (53 %) also utilized *in vitro* assays to evaluate the drugs antileishmanial effect (in 7 studies, *in vitro* assays were performed before *in silico*), 13 reported IC/EC_50_ values (4 using MTT, 5 Cc, 3 alamarBlue (resazurin), 1 HIA, 1 Fc), 2 reported cell viability percentages (1 MTT and 1 Cc), 1 reported parasite load on macrophages (by kDNA expression), 1 reported percentage of inhibition at 5 µM (compared to AmphB), and in 1 study the only *in vitro* assay was related to another protozoan. A total of 43 drugs were tested in these studies, against *L. donovani* (n=22), *L. amazonensis* (n=11), *L. braziliensis* (n=10), *L. mexicana* (n=9), *L. panamensis* (n=5), *L. martiniquensis* (n=2), and *L. major* (n=1). Of these, 37 were evaluated on promastigotes and 20 on intracellular amastigotes (14 on both forms). Luliconazole, a topical azole antifungal agent, exhibited the highest potency among all studies, with an IC_50_ of 0.07 μM against *L. major* intracellular amastigotes. 

Arguably, flow cytometry is a more complete technique to evaluate leishmanicidal activity compared to colorimetric assays (alamarBlue, MTT), allowing the evaluation of intracellular amastigotes and cell-cycle markers. Unfortunately, it is significantly more expensive in terms of equipment and reagents. Direct cell counting, in contrast, is time-consuming and error-prone due to relying on operator experience and bias. New techniques involving reporter genes, transgenic *Leishmania*, high-content imaging, and ex vivo may also be used for fast and reliable screening of drugs (Fumarola et al., 2004[63]; Zulfiqar et al., 2017[228]).

Notably, 10 drugs exhibited poor leishmanicidal activity *in vitro*: refametinib, binimetinib, selumetinib, tenofovir, lamivudine, metformin, pioglitazone, zafirlukast, cyproheptadine, and nilotinib. Interestingly, almitrine and midostaurin were effective against *L. amazonensis* (IC_50_ < 3 µM) but displayed no inhibition over *L. donovani*, suggesting a species-specific activity. Source information is available in Supplementary data, Supplement 4.

### Antileishmanial targets and drugs: Perspectives stemming from in silico experiments

**Figure 5:**
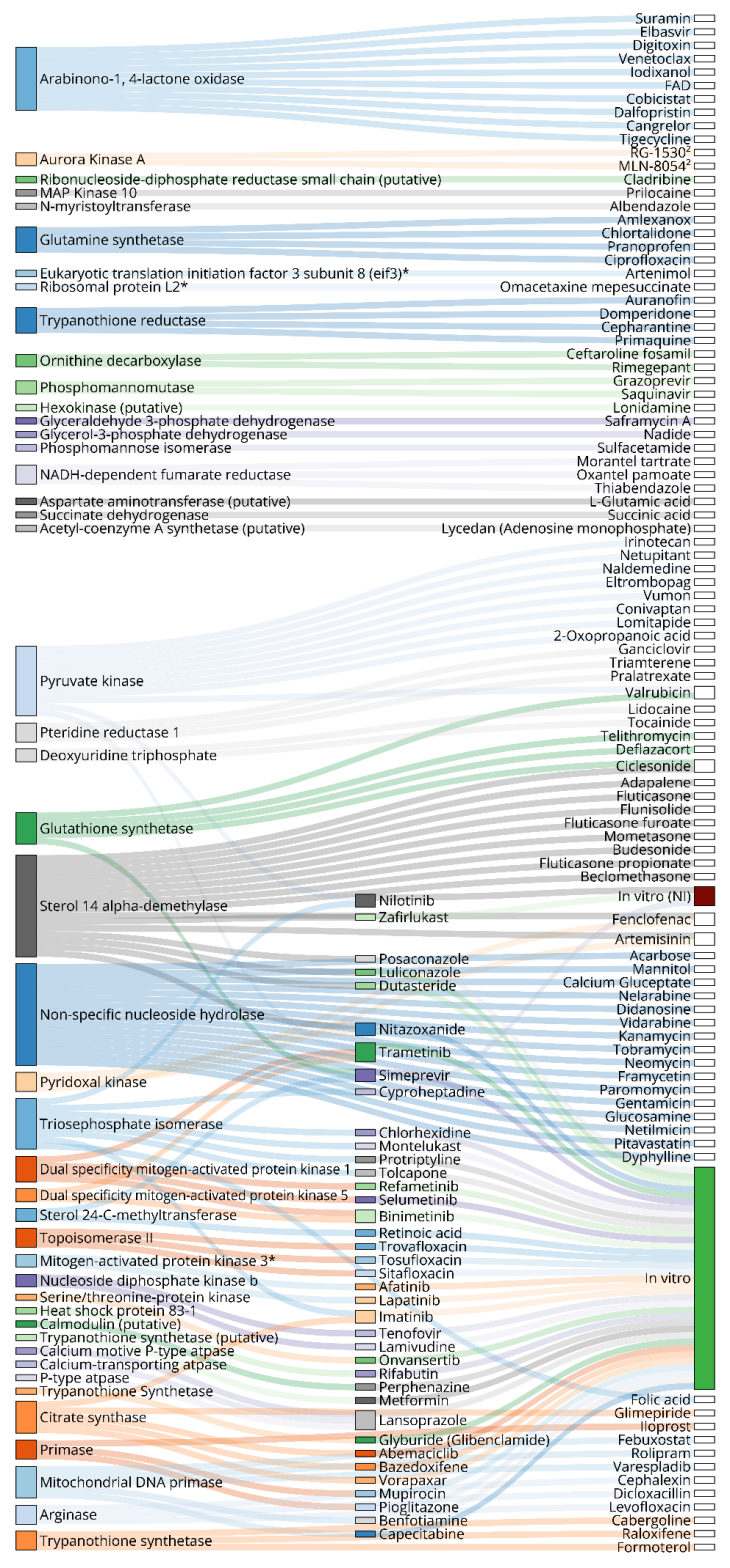
Sankey plot representing ligand-target relationship and *in vitro* testing of drugs. Targets are presented on the left and drugs evaluated *in vitro* in the middle. On the right, ligands that were only tested *in silico*. The number of ligand-target associations is proportional to the width of the respective nodes. Colors are provided for better visualization only.

A total of 94 targets (72 unique) were directly reported (high-throughput methods that presented over 20 targets were not considered in this count), and 45 targets were associated with a total of 151 potential ligands (154 unique ligands in total). In terms of species, the majority of the targets were from *L. major* (n=43; 48 %), followed by *L. donovani* (n=18; 20 %), *L. infantum* (n=12; 13 %), *L. braziliensis* (n=11; 12 %), *L. mexicana* (n=5; 5.5 %), and *L. amazonensis* (n=1; 1 %). The most frequent targets were sterol 14-alpha demethylase (n=5) and trypanothione reductase (n=5), followed by glycerol-3-phosphate dehydrogenase and trypanothione synthetase (n=3), ornithine decarboxylase, pteridine reductase 1, P-type ATPase, alcohol dehydrogenase (putative), sterol 24-C-methyltransferase, ribonucleoside-diphosphate reductase, primase, arginase and phosphomannose isomerase, appeared in 2 studies each. Other targets were reported once. Targets and associated ligands are presented as a Sankey plot in Figure 5(Fig. 5).

The targets associated with the highest number of ligands were: NNH (16 ligands), SDM and SMT (15 ligands, each), PK (n=11 ligands), and ALO (n=10 ligands). The lowest free binding energy for a ligand-target complex was for amlexanox-Glutamine synthetase (-221.68 kcal/mol), using the MM/PBSA method. For studies that only reported a docking binding energy, the best result was for dutasteride-SDM (-11.7 kcal/mol in Vina), dicloxacillin-Arginase (-124.112 kcal/mol in Moldock), and omacetaxine mepesuccinate-RPL2 (-40.706 kcal/mol in Glide). Binding energies for all reported complexes are summarized in Supplementary information, Supplement 5. Interestingly, dutasteride displayed a potent antileishmanial activity *in vitro*, with an IC_50_ of 1.25 µM against *L. donovani* intracellular amastigotes and induced an apoptotic cell-death caused by ROS production (Tabrez et al., 2021[195]). It is important to note that docking-based affinity prediction is not highly accurate, and scoring values cannot be directly compared between different software (Pantsar and Poso, 2018[140]). Nine enzymes were chosen for discussion based on the highest frequency of studies or the number of associated ligands: TryR, SDM, PK, TIM, CS, GS, NNH, and ALO. In addition, structures, subcellular location, and involved pathways of 11 targets are presented in Figure 5(Fig. 5). Source information is available in Supplementary information, Supplement 6.

#### Trypanothione reductase

**Figure 6:**
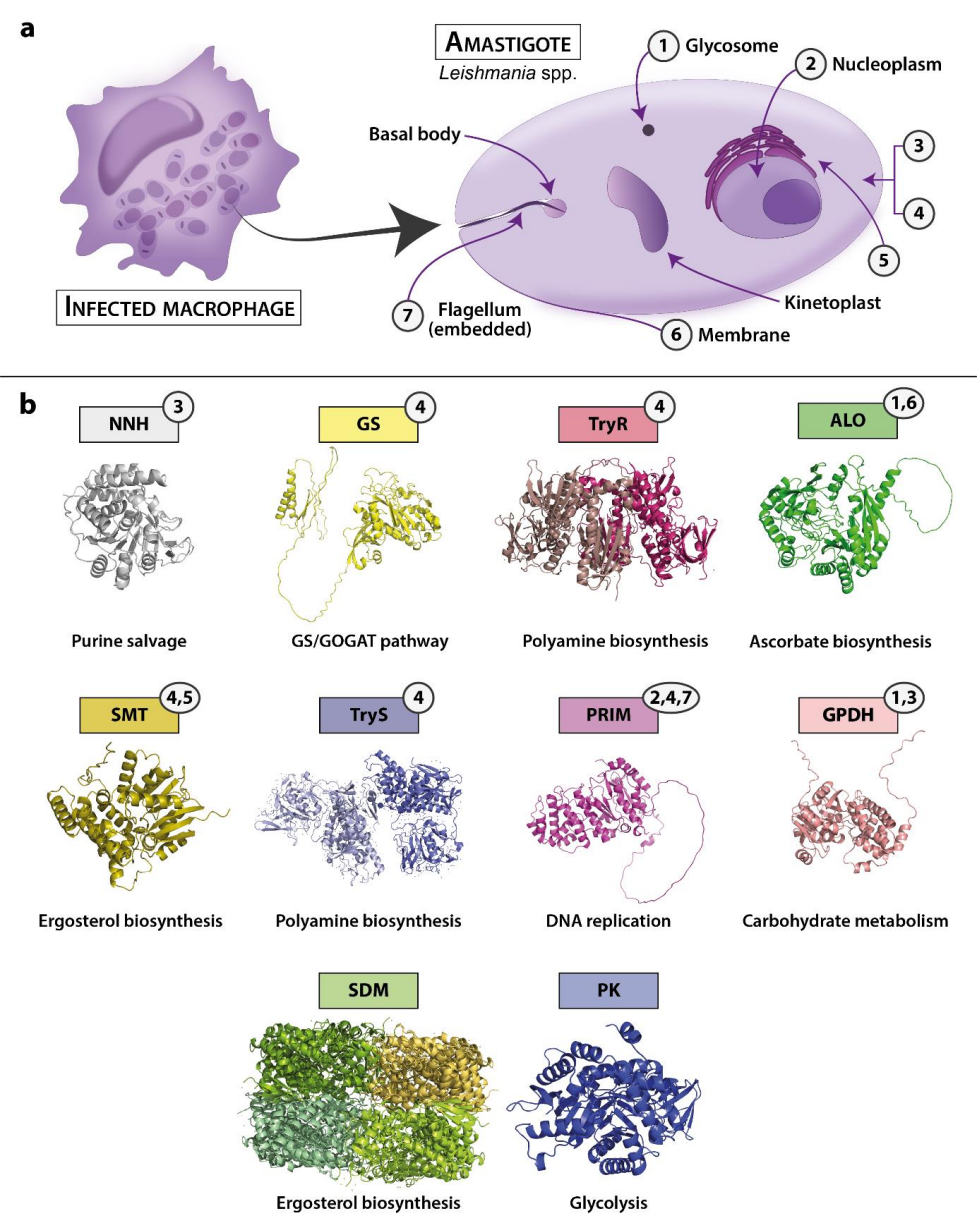
Frequent in silico targets in included studies and their subcellular localization. (A): Schematic representation of an infected macrophage (left) and an amastigote form containing relevant cellular components. (B): Tridimensional structures and pathways of targets found in the literature, selected based on the frequency of studies and/or number of promising hits in silico. Proteins are represented in cartoon, each with distinct colors. For TryS and TryR, chains A and B are presented in different shades. Numbers indicate their subcellular location, considering Gene Ontology (GO) prediction obtained from UniProt. Flagellum is used herein as equivalent for the GO term “ciliary plasm.” 3, 4, and 5: Cytosol, cytoplasm, and endoplasmic reticulum, respectively. NNH = Nonspecific nucleoside hydrolase, GS = Glutamine synthetase, TryR = Trypanothione reductase, ALO = Arabinono-1,4-lactone oxidase, SMT = Setrol C-24 methyltransferase, TryS = Trypanothione synthetase, PRIM = DNA primase, GPDH = Glycerol-3-phosphate dehydrogenase, SDM = Sterol 14-α demethylase, PK = Pyruvate kinase.

Trypanothione reductase (TryR) (Figure 6(Fig. 6)) is a homodimeric NAPDH-dependent flavoprotein disulfide reductase enzyme presented in a variety of organisms, including *Leishmania *spp., being a potential therapeutic target used in the discovery of new therapies since it is absent in humans (Battista et al., 2020[17]; Madia et al., 2023[105]). TryR is fundamental in protecting parasite cells against oxidative damage by regenerating trypanothione (TS2), a dithiol formed by two glutathione molecules connected by spermidine (polyamine). The antioxidant metabolism is also related to the differentiation process from promastigotes to amastigotes within the parasitophorous vacuole of the invaded host cell macrophage, which releases reactive oxygen and nitrogen species as cellular defense (Marchese et al., 2018[108]; Reverte et al., 2021[159], 2022[160]). Various structures of leishmanial TryR have been experimentally obtained, containing antimony (PDB ID: 2W0H), auranofin (2YAU), selective inhibitors (6T97, 6T98, 4APN), and its native ligand (4ADW). Reduction of TS2 occurs in the deeply buried dual-catalytic site formed at the dimer interface, in which two cysteine residues, Cys52 and 57, interact with sulfur atoms from the substrate (Baiocco et al., 2013[14]). At least two druggable sites are well-characterized: The mepacrine binding site (1), located at the entrance of the catalytic site, promotes blockage of TS2 access and induces conformational changes in the enzyme, and the NADPH binding cavity (2), resulting in competitive inhibition with this cofactor (Battista et al., 2020[17]). The large volume of the active site constitutes an additional difficulty for docking-based screening, resulting in unspecific binding poses (Beig et al., 2015[19]).

Several recent studies on TryR inhibitors using the structure-based virtual screening (SBVS) approach were conducted with both repurposed drugs and derivatives of natural products. For example, a flavonoid found in green tea, (-)-Epigallocatechin-3-O-gallate, inhibited the proliferation of *L. infantum* promastigotes, acting as a competitive TryR inhibitor ( % inhibition = 95.8) (Inacio et al., 2021[80]). Phenothiazine derivatives, including chlorpromazine, are potent inhibitors of TryR and amastigotes forms (Chan et al., 1998[35]).

In this review, five articles (15 %) explored drug repositioning targeting TryR from *Leishmania *(Chavali et al., 2012[36]; Waugh et al., 2014[213]; Silva et al., 2015[190]; Abhishek et al., 2019[1]; Harigua-Souiai et al., 2022[73]). Chavali et al. (2012[36]) identified 254 FDA-approved ligands with known targets similar to trypanosome reductase using the Metabolic Network Guided Drug Pipeline (MetDP), predicting the activity of nitrofurazone and nitrofurantoin on TryR from *L. major* (gene LmjF06.0860). Recent studies show that synthetic derivatives of both drugs exhibit multi-target inhibition and have demonstrated *in vitro* activity against trypanosomatids (Santiago et al., 2020[173]; Scarim et al., 2021[177]; Seetsi et al., 2024[180]). Ndlovu et al. (2023[131]) found two ethylene glycol derivatives of nitrofurantoin as anti-promastigote leads, with drug-like properties, and *in vitro* inhibition of *L. donovani* (IC_50 _0.78 ± 0.09 µM and 0.73 ± 0.16 µM) and *L. major *(IC_50_ 0.19 ± 0 µM and 0.27 ± 0.01 µM), and reduction of the growth of these parasites by 88 % and 72 %, respectively, but without significant results for anti-amastigote activity. For *L. donovani*, Zuma et al. (2023[229]) also found *in vitro*, using THP-1 host cells, a nitrofurantoin-derivative lead compound (with a n-pentylene linker) with promastigote cytotoxicity (IC_50_ >100 µM), but also not exhibiting anti-amastigote properties (Ndlovu et al., 2023[131]; Zuma et al., 2023[229]).

Waugh et al. (2014[213]) compiled a list of prospective candidates for various targets, identifying primaquine as a TryR inhibitor for *L. major* with five pharmacophoric features (AUC = 0.9). Primaquine, an 8-aminoquinoline used in malaria, is a pro-drug effective against multiple *Plasmodium* spp. However, its mechanism of action remains unclear, and it is unsafe for individuals deficient in glucose-6-phosphate dehydrogenase (G6PD) due to hemolytic toxicity (Ashley et al., 2014[11]). Derivatives of primaquine and 8-aminoquinolines have been synthesized and evaluated for *Leishmania* spp. For example, two peptidomimetic and organometallic derivatives of primaquine, in an *in vitro* study of *L. infantum* by Vale-Costa et al. (2012[203]) showed low toxicity in host cells compared to sitamaquine and miltefosine, with a reduction of over 96 % in intracellular parasitic load, serving as prototypes for new hit compounds for visceral leishmaniasis.

Silva et al. (2015[190]) also proposed cepharanthine as an *L. major* TryR inhibitor (LmjF05.0350) in their multi-target study but without data on toxicity and druggability. Cepharanthine, the only bisbenzylisoquinoline alkaloid approved for human use, exhibits anti-inflammatory, antineoplastic, antioxidant, and anti-parasitic properties by modulating cell membranes, activating AMPK, and inhibiting the NF-κB signaling pathway (Bailly, 2019[12]; Liu et al., 2023[101]). In other studies, with *T. cruzi*, its inhibition of trypanothione reductase had previously been observed, and a significant increase in cases of negative serology and the number of survivors of mice with chronic *T. cruzi* infection (p < 0.05), but not in acute cases was observed in a study of Fournet et al. (2000[60]).

Abhishek et al. (2019[1]) demonstrated that auranofin (and its intermediates), an approved drug for rheumatoid arthritis, could effectively inhibit TryR of *L. infantum* and interfere with the parasite's ROS metabolism and thiol-based redox balance. Auranofin is positioned near the two catalytic Cys residues, forming a coordinated adduct that permanently inhibits TryR function. Specifically, Glu467, Ser470, and His461 engage in hydrogen bond or ionic interactions, while Phe396, Lys61, and Pro462 establish hydrophobic contacts with the ligand. Additionally, the hydrophobic regions of the TP moiety of auranofin interact through van der Waals or hydrophobic interactions with the side chains of Thr335 and His461 residues. Auranofin is highly thiophilic and demonstrated *in vitro* inhibitory activity against TryR (Ilari et al., 2012[79]; Sharlow et al., 2014[186]), and in a recent study by Berneburg et al. (2023[22]), auranofin inhibited6-Phosphogluconate Dehydrogenase (6PGD) from *L. donovani* (IC_50_ 8.6 ± 1.0 µM). 6PGD is involved in the pentose phosphate pathway (PPP), reducing NADP^+^ to NADPH and thus capable of influencing cellular redox homeostasis (Berneburg et al., 2023[22]). Silver can also inhibit TryR via coordination with catalytic Cys residues (Baiocco et al., 2011[13]).

Harigua-Souiai et al. (2022[73]) identified 14 ligands for *Leishmania *spp. through ML, revealing that domperidone could inhibit *L. infantum* TryR. Domperidone, a benzimidazole derivative and dopamine receptor (D2) antagonist, accelerates gastrointestinal peristalsis and prolactin release and has been evaluated for canine VL as immunotherapy, exhibiting promising results in clinical trials (Gómez-Ochoa et al., 2009[67]; Sabaté et al., 2014[166]; Baxarias et al., 2023[18]; Yıldırım et al., 2023[224]). It should be noted that none of the articles explored TryR in an extensive docking-based VS campaign with approved compounds.

#### Trypanothione synthetase

Trypanothione synthetase (TryS) is a ligase enzyme found in certain parasitic organisms, especially those belonging to the *Trypanosomatidae* family. Its primary function is associated with thiol redox metabolism, catalyzing the synthesis of trypanothione, which plays a pivotal role in shielding the parasite from oxidative stress, a crucial factor for its survival and infectivity. TryS presents two domains with distinct activities: The N-terminal domain, with amidase activity, not related to growth and infectivity, and the C-terminal domain, responsible for synthetase activity and essential for thiol formation. Given its exclusive presence in parasitic organisms, TryS emerges as a promising target for developing antiparasitic drugs with high specificity and minimal toxicity to the host (Rub et al., 2019[164]; Phan et al., 2022[146]; Ihnatenko et al., 2023[78]). *In silico* evaluation involving TryR and TryS, Mehwish et al. (2019[110]) demonstrate that some natural compounds, such as quercetin, gallic acid, rutin, and lupeol, can inhibit both enzymes in amastigote and promastigote forms of *L. tropica*, with *in vitro* results suggesting reduced parasite cell growth.

This review included three articles (9 %) (Bustamante et al., 2019[29]; Rub et al., 2019[164]; Vemula et al., 2024[207]). Bustamante et al. (2019[29]) targeted TryS (putative) from *L. braziliensis* using a PPI network and identified metformin as a repurposable candidate, presenting low toxicity *in vitro* (LC_50_ > 200 μg/mL). One potential application of this hypoglycemic drug in leishmaniasis is to interfere with glucose absorption metabolism, which can impact the growth and survival of parasites. However, Lima et al. (2020[100]), who evaluated the immunomodulatory effects of metformin *in vitro* using raw macrophages (ATCC 264.7) and *in vivo* with BALB/c mice infected with *L. braziliensis*, observed that metformin increased the intracellular viral load of macrophages and the viability of the parasite in the parasitophorous vacuole *in vitro* (p = 0.006, and p = 0.02, respectively). They also noted a 1,000-fold increase in parasites at the inoculation site and in the lymph nodes, suggesting that metformin alters oxidative stress metabolism but reduces the antimicrobial activity of macrophages.

Rub et al. (2019[164]) evaluated glyburide (glibenclamide) against TryS from *L. donovani* promastigotes using molecular docking, which showed a binding affinity of -7.6 kcal/mol. In *in vitro* experiments, the authors demonstrated that a 40 μg/mL dose significantly inhibited the growth of approximately half of the parasites (p < 0.01), and also affected their cellular morphology. Glibenclamide, a second-generation sulfonylurea, is an oral antidiabetic drug in type II diabetes mellitus. It functions as an ATP-sensitive K^+^ channel inhibitor and a selective blocker of ABC transporters, capable of transporting leishmanicidal compounds out of the cellular environment. Additionally, glyburide can synergize with other antiparasitic treatments against *Leishmania *spp., enhancing the efficacy of treatments such as glucantime Sb (V), particularly in promastigote forms that are known to be resistant to pentavalent antimonials in solution (Padrón-Nieves et al., 2009[138]). However, further studies are still needed to properly evaluate this correlation. A study evaluating patients with cutaneous leishmaniasis (CL) showed that glibenclamide can reduce local inflammation dose depending (Carvalho et al., 2020[33]). This reduction is achieved by downmodulation of the production of the pro-inflammatory cytokine, including IL-1B, IL-17, and TNF (p < 0.0001, p < 0.001, and p < 0.05, respectively) when 200 µL of glyburide is associated with soluble *Leishmania *antigen. However, no reduction in parasitemia in macrophages was observed after treatment.

Vemula et al. (2024[207]) screened the FDA-approved drugs dostinex (cabergoline, dopaminergic D2 agonist), raloxifene (selective estrogen receptor modulator), and formoterol (selective β₂ agonist) as inhibitor candidates of TryS from a homology model based on *L. major* (2VOB). These drugs demonstrated good affinity and stability with the receptor, as evidenced by the dock scores, -11.927 kcal/mol, -10.568 kcal/mol, and -10.446 kcal/mol, respectively, and the binding energy of the complexes -56.21 kcal/mol, -70.41 kcal/mol, and -64.15 kcal/mol, respectively. Dostinex exhibited toxicity in class 3 (LD_50_ = 200 mg/kg), raloxifene in class 4 (LD_50_ = 400 mg/kg), and formoterol in class 5 toxicity (LD_50_ = 3130 mg/kg). Among these compounds, Reimão et al. (2014[157]) previously evaluated raloxifene for treating leishmaniasis, both *in vitro* and *in vivo*. They investigated its mechanism of action against various species of *Leishmania *in both promastigote and amastigote forms (with an EC_50_ of 15.0 ± 2.3 µM for *L. amazonensis*). Structural changes were observed in both cellular forms of parasites but without disruption of the cell membrane. In experiments with BALB/C mice infected with *L. amazonensis*, a reduction of 54.3 % in lesion size was observed in the raloxifene group after five weeks of treatment (100 mg/kg/day).

#### Sterol 14 α-demethylase

Sterol 14 α-demethylase (SDM) (Figure 6(Fig. 6)), also referred to as CYP51, is an enzyme belonging to the cytochrome P450 superfamily, involved in sterol biosynthesis in eukaryotes (Zhang et al., 2019[226]). Within fungi, this enzyme serves as a critical target for azoles (e.g., ketoconazole, fluconazole, itraconazole, voriconazole, posaconazole, and isavuconazole). These antifungals work by inhibiting CYP51 activity, decreasing ergosterol production, and disrupting membrane integrity (Emami et al., 2017[52]). In *Leishmania*, SDM catalyzes the conversion of lanosterol to ergosterol, a cell membrane structural component, contributing to its fluidity and stability. Additionally, this sterol regulates membrane-bound proteins and ion channels, influencing various cellular processes. The inhibition of this enzyme can lead to severe membrane defects and impairment of essential cellular functions, compromising the integrity of the membrane and making it more susceptible to environmental stressors and host immune responses (Mwenechanya et al., 2017[128]; Mukherjee et al., 2020[121]; Karamysheva et al., 2021[85]).

In this present review, five studies (15 %) that analyzed *in silico* inhibitors of SDM were included (Chavali et al., 2012[36]; Shokri et al., 2018[189]; Tabrez et al., 2021[195]; Gupta et al., 2022[70]; Saha et al., 2023[168]). Chavali et al. (2012[36]) compared the drug predictions accordingly with literature findings, identifying seven antifungal drugs, in addition to amphotericin B, as true positives: ketoconazole, fluconazole, clotrimazole, itraconazole, metronidazole, miconazole, and terbinafine. In this context, another included study by Gupta et al. (2022[70]) also evaluated azoles against *Leishmania*. It concluded that posaconazole could also be repurposed for leishmaniasis by inhibiting the sterol 14 α-demethylase, with verified binding stability by induced fit docking and MD simulations, and *in vitro* activity against *L. donovani* amastigotes (IC_50_ = 1.64 ± 0.37 µM). MD revealed that Phe289 and Glu100 are important to stabilizing tetrahydrofuran and alkoxy phenyl moieties, with pi-stacking interactions involving Tyr102, Phe104, Tyr115, and aromatic rings. Interestingly, this binding mode is in accordance with an experimental structure of SDM with the azole portion of fluconazole interacting with heme iron (Hargrove et al., 2011[72]). Saha et al. (2023[168]) also verified the importance of Tyr115 *in silico* for ligand binding. Azoles have been evaluated in monotherapy and as an adjunct therapy to other antiparasitic drugs for leishmaniasis, especially CL. A recent study by Paul et al. (2024[142]) assessed the activity of clotrimazole *in vitro* against promastigotes, intracellular amastigotes, and macrophages infected with *L. donovani* (IC_50_ = 35.75 ± 1.06 μM, 12.75 ± 0.35 μM, and 73 ± 1.41 μM, respectively). A dose-dependent inhibition was observed, with clotrimazole being 5.73 times more selective against amastigotes compared to host cells, and significant membrane depolarization was induced in *L. donovani* promastigotes treated with clotrimazole compared to the miltefosine-treated and untreated groups (p < 0.001). Despite ongoing controversy regarding the efficacy of these medications for leishmaniasis, as reported with fluconazole in clinical trials (Emad et al., 2011[51]; Prates et al., 2017[150]; Parhizkar et al., 2024[141]), assessing the synergistic effect of these drugs and their derivatives with other leishmanicidal therapies and different dose regimens is recommended, also considering the potential toxicity of azoles at higher doses.

Terbinafine is a widely safe antifungal medication from the allylamines class, which interferes with growth and viability by inhibiting squalene-2,3-epoxidase and depleting sterols. However, according to a systematic review conducted by Bezemer et al. (2021[23]), there is no evidence to support the efficacy of terbinafine for mucocutaneous leishmaniasis. Nevertheless, further studies evaluating its synergistic effect, especially with azoles, should be conducted based on the results of *in vitro* studies assessing combinations with other candidates and leishmanial drugs.

While the complete mechanism remains unclear, luliconazole, a novel topical antifungal imidazole, demonstrates effectiveness against dermatophyte organisms, including azole-resistant strains. Similar to allylamines, imidazoles also inhibit ergosterol synthesis and other pathways by inhibiting cytochrome P450 (CYP450), potentially leading to cell necrosis due to hydrogen peroxide formation. Shokri et al. (2018[189]) are the only ones to date to evaluate luliconazole for leishmaniasis through *in silico* studies in *L. infantum* (PDB code: 3L4D) and *in vitro* with amastigote and promastigote forms of *L. major*, compared to other therapies such as ketoconazole, meglumine antimoniate, and amphotericin B. A docking simulation was performed to better understand the interactions between luliconazole and sterol 14 α-demethylase, revealing several hydrophobic interactions and coordination with the heme iron of the porphyrin prosthetic group of the enzyme. In the anti-promastigote activity assay, luliconazole showed a significant reduction in the viability of promastigotes, with an IC_50_ value (IC_50_ 0.19 μM) significantly lower than the other therapies tested (IC_50_ meglumine antimoniate = 538 μM, IC_50_ ketoconazole = 135 μM, and IC_50_ amphotericin B = 2.52 μM). In amastigotes, the imidazole candidate was less toxic than the controls and significantly decreased the mean infection rate and the mean number of amastigotes per macrophage (p < 0.001) compared to ketoconazole and meglumine antimoniate, but not in relation to amphotericin B (p > 0.05).

Some treatments for other parasitic diseases have been studied for cutaneous and visceral leishmaniasis. Saha et al. (2023[168]), using docking approaches, MD simulations, MMPBSA, and binding free energy analysis for sterol 14 α-demethylase and pyridoxal kinase targets from *L. donovani*, found that nitazoxanide, artemisinin, and fenclofenac (a discontinued nonsteroidal anti-inflammatory drug) showed potential antileishmanial activity (docking scores: -7.58 kcal/mol for nitazoxanide, -7.95 kcal/mol for artemisinin, and -7.39 kcal/mol for fenclofenac), with nitazoxanide showing the most promising results regarding stability and structural preservation of the target protein (MMPBSA energy = 175.61 ± 12.64 kJ/mol). Nitazoxanide (NTZ) is a synthetic nitrothiazolyl-salicylamide derivative a broad-spectrum treatment, and the first-line option for Cryptosporidium parvum and Giardia lamblia infections. In addition to the described inhibition of the sterol 14 α-demethylase for *Leishmania *spp., in diseases caused by protozoa, NTZ is also capable of altering the polarity of mitochondrial membranes, and influencing cellular energy metabolism and detoxification, with inhibition of the pyruvate: ferredoxin/ flavodoxin oxidoreductase (PFOR) and other enzymes such as quinone oxidoreductase NQO1, nitroreductase-1, glutathione-S-transferase, and protein disulphide isomerase enzymes (Shakya et al., 2018[183]). New nanoliposomal formulations are being used to improve drug bioavailability and delivery. Pinto Torres et al. (2020[147]) found that liposomes containing nitazoxanide showed good efficacy *in vitro* (IC_50_ = 16 μM in amastigotes), and *in vivo* against *L. infantum* forms, reducing the parasite burden in the liver and spleen (p < 0.05), what makes it a candidate for the treatment of visceral leishmaniasis.

#### Pyruvate kinase

The pyruvate kinase (PK) (Figure 6(Fig. 6)) is a homotetrameric and allosteric enzyme involved in glycolysis - a process responsible for energy generation by the irreversible conversion of phosphoenolpyruvate and ADP to pyruvate and ATP, with fructose 2,6-bisphosphate as an allosteric activator, and K^+^ and Mg^2+^ as cofactors, influencing essential cell processes and being particularly crucial in cells with high energy demands (Pinto Torres et al., 2020[147]; Schormann et al., 2019[179]). These parasites can be found in low oxygen conditions in host macrophages, where anaerobic glycolysis is crucial, especially in amastigote form. Moreover, the activity of pyruvate kinase can significantly contribute to the ability of *Leishmania *cells to adapt to these conditions and survive within the intracellular environment of the host (Degrossoli et al., 2011[48]; Conceição-Silva and Morgado, 2019[41]; Ohms et al., 2021[134]; Pawłowska et al., 2023[143]). Interestingly, *L. mexicana* and *T. cruzi* pyruvate kinases were previously crystallized with a glycolysis inhibitor, suramin (PDB ID: 3PP7), occupying the adenosine binding site (Morgan et al., 2011[118]). In *Trypanosoma brucei*, suramin has been characterized to inhibit seven other enzymes involved in glycolysis (Willson et al., 1993[217]), and an RNAi experiment confirmed the lethality of inhibition of PK in this trypanosomatid (Coustou et al., 2003[42]). It should be noted that the trypanocidal effect of suramin could stem from a multi-target interaction. Although its mechanism of action has not been completely elucidated yet, inhibition of glycolytic enzymes is well characterized, but they are unlikely to be the primary targets (Jaffe et al., 1972[81]; Vansterkenburg et al., 1993[204]; Wiedemar et al., 2020[216]; Zoltner et al., 2020[227]).

Only two studies (6 %) evaluating *in silico* potential inhibitors of pyruvate kinase (PK) were identified in this review (Silva et al., 2015[190]; Amiri-Dashatan et al., 2021[6]). Amiri-Dashatan et al. (2021[6]) identified 10 inhibitors and three promising candidates for further studies based on their tolerability profiles, which included nilotinib - a tyrosine kinase inhibitor used in certain types of chronic myeloid leukemia (dock score: -10.1kcal/mol), netupitant - an antiemetic (dock score: -10.1 kcal/mol), and conivaptan - a non-peptide inhibitor of vasopressin, approved for hyponatremia caused by the syndrome of inappropriate antidiuretic hormone (SIADH) (dock score: -9.9 kcal/mol). The binding site corresponded to the one Willson et al. (1993[217]) determined, encompassing Pro29, His54, and Arg49 residues. In contrast, Juarez-Saldivar et al. (2023[83]) reported no inhibition of nilotinib over *L. mexicana* *in vitro*. Enzymatic inhibition assays demonstrated that two antiparasitic drugs, nitazoxanide, and niclosamine, are active against *L. mexicana* PK (assay ChEMBL ID 1614285 and 1613913). 

#### Triosephosphate isomerase

Triosephosphate isomerase (TIM) is a non-allosteric and dimeric enzyme present in various organisms, including mammals and parasites. It is involved in the initial reactions of glycolysis by the reversible conversion of dihydroxyacetone phosphate and glyceraldehyde-3-phosphate through an intermediate enediol. It can also affect other pathways, such as gluconeogenesis, and fatty acid biosynthesis. This enzyme is found in the cytosol and in organelles called glycosomes, which are exclusive to kinetoplastids, presenting structural similarity among parasites such as *T. cruzi, T. brucei*, and *L. mexicana*, but dissimilar to other species like humans, thus conferring an advantage as a therapeutic target (Olivares-Illana et al., 2007[135]; Vázquez-Jiménez et al., 2023[206]). Rational selection of benzimidazole derivatives based on docking scores against *L. mexicana* TIM resulted in the identification of a potent leishmanicidal (IC_50_ = 4.04 µM) (Vázquez-Jiménez et al., 2023[206]). TIM was also studied for immunization against VL and induced protective immunity in around 90 % of vaccinated animals (Kushawaha et al., 2012[98]).

In this review, two studies (6 %) identified possible leishmanicidal candidates for TIM inhibition by *in silico* approaches (Chavali et al., 2012[36]; Juarez-Saldivar et al., 2023[83]). Chavali et al. (2012[36]) identified 254 FDA-approved ligands with 10 known targets using the Metabolic Network Guided Drug Pipeline (MetDP). For TIM, they found aspartame (artificial sweetener), sucralfate (antiacid), ipratropium (bronchodilator), trifluridine (antiviral), framycetin (antibiotic), quinacrine (antimalarial compound), and pyruvic acid (organic compound) with a 0.8 druggability index (based on similarity to the known biological target). Quinacrine is an acridine derivative similar to 4-aminoquinolines and has been used for over a century in the prevention and treatment of malaria, especially during World Wars. However, there is a noted incidence of severe adverse events, such as methemoglobinemia and hemolysis, which compromises its use. Nevertheless, due to its high potency against *Plasmodium* spp. forms, it has been a scaffold for synthesizing more tolerable derivatives (Fonte et al., 2021[59]; Shanks, 2022[184]). For *Leishmania *spp., Wong et al. (2009[218]) noted that quinacrine can reverse pentamidine resistance, depending on the administered dose (6 μM), reducing the IC_50_ of pentamidine by 4.2-fold for *L. enriettii* pentamidine-resistant (IC_50_ of pentamidine = 224 μM) and 1.9-fold for *L. donovani* pentamidine-resistant type (IC_50 _of pentamidine = 74 μM).

Juarez-Saldivar et al. (2023[83]) virtually screened repositioning drugs with dual inhibition potential through structure-based virtual screening and molecular docking against triosephosphate isomerase of *T. cruzi* (TcTIM, PDB: 1SUX) and *L. mexicana* (LmTIM, PDB: 1AMK). They identified eight compounds: chlorhexidine (-8.9 kcal/mol), cyproheptadine (-8.2 kcal/mol), folic acid (-7.6 kcal/mol), imatinib (-8.2 kcal/mol), montelukast (-7.6 kcal/mol), nilotinib (-7.6 kcal/mol), protriptyline (-7.5 kcal/mol), and tolcapone (-7.8 kcal/mol). From the *in vitro* evaluation, not performed for imatinib, the authors found that chlorhexidine and protriptyline are the most active against *T. cruzi* trypomastigotes and *L. mexicana* promastigotes (IC_50_ of chlorhexidine = 1.73 ± 0.5 μM, and IC_50_ of protriptyline = 1.65 ± 0.09 μM). For chlorhexidine, hydrogen bonds with Gln112(A), Ala70(A), Tyr103(B), hydrophobic bonds with Ile109(A), Lys71(A), and Phe75(A), and cation-pi interactions with Phe75(A) residue were observed. As for protriptyline, there were hydrogen bonds with Glu105(A), hydrophobic bonds with Ile109(A), Ile69(A), Ala70(A), Phe75(A), Tyr102(B), and Tyr103(B), and pi stacking with the Phe75(A) in the interface of the binding site.

Chlorhexidine is a cationic bis-biguanide formed by two 4-chlorophenyl rings and two biguanide groups, united by a central hexamethylene chain. This compound is a broad-spectrum antiseptic and disinfectant, commonly found in topical formulations and oral rinses for dental care (Mohammadi and Abbott, 2009[116]), with antiparasitic activity still largely unexplored. From the chlorhexidine molecule, newly derived compounds, more efficient yet equally potent, have shown inhibitory activity against *T. cruzi* TryR, which may confer to this compound a multi-target ability against trypanosomatids that can be further explored (Meiering et al., 2005[111]).

Protriptyline is a tricyclic antidepressant (TCA) mainly used to treat major depressive disorder (MDD). It primarily inhibits serotonin and norepinephrine reuptake and antagonizes muscarinic cholinergic, histamine (H1), and alpha-1 receptors, as well as sodium and calcium cardiac channels. In other studies, clomipramine was able to selectively inhibit both extracellular and intracellular forms of *L. amazonensis* *in vitro* (IC_50_ of 8.31 ± 3.29 μM and IC_50_ of 5.45 ± 4.92 μM, respectively), significantly inducing (p < 0.05) oxidative stress, morphological alterations, and apoptosis (da Silva Rodrigues et al., 2019[45]). In a Phenotypic Screening Assay conducted by Alcântara et al. (2020[5]), several molecules were simultaneously screened for *Leishmania *spp., and protriptyline, along with other compounds, exhibited pan-leishmanial activity in THP-1 cells, with 100 % maximum activity against *L. amazonensis*, 82 % against *L. braziliensis*, and 58 % against *L. donovani*.

#### Citrate synthase

Citrate synthase (CS) (Figure 6(Fig. 6)) is a mitochondrial enzyme that catalyzes the conversion of acetyl-CoA and oxaloacetate into citrate, marking the initial step of the citric acid cycle, also known as the Krebs cycle or tricarboxylic acid (TCA) cycle. This set of reactions is intricately linked to the electron transport chain and is a crucial component of aerobic energy production (Chhimpa et al., 2023[39]). While the potential of CS as an antileishmanial drug target is unclear, a thermal shift experiment with miltefosine-treated *L. infantum* suggests some degree of interaction (Ibarra-Meneses et al., 2022[77]). Interestingly, inhibition of early steps of the TCA cycle is deleterious in amastigotes by limiting available α-ketoglutarate for glutamate synthesis, which is necessary for thiol metabolism. Additionally, amastigotes have a reduced glutamate uptake capacity (Saunders et al., 2014[176]).

Ranjan and Dubei (2023[154]) discovered three inhibitors of *L. donovani* promastigote and intracellular amastigotes after virtual screening against CS: Abemaciclib, bazedoxifene, and vorapaxar, with abemaciclib, an antineoplastic agent used in advanced breast cancer, identified as the most promising candidate, showing hydrophobic interaction with Ala243, Ser251, Ala277, His281, Gly282, Leu283, and Gln286 residues, and H-bond interaction of pyridine with His242, and piperazine groups with Tyr238 and Asn285, exhibiting a low binding energy value (binding energy = -10.22 kcal/mol), low predicted toxicity (CC50 against J774A.1 cell lines = 83.35 ± 0.77 μM) and good *in vitro* activity for further *in vivo* studies (IC_50_ value against promastigote = 0.92 ± 0.02 μM, EC_50_ value against amastigote = 1.52 ± 0.37 μM).

#### Glutamine synthetase

Glutamine synthetase (GS) (Figure 6(Fig. 6)) is an Mn-dependent enzyme involved in nitrogen metabolism, playing a crucial role in glutamine synthesis. This essential amino acid has various functions, such as protein and nucleic acid synthesis, ammonia transport, regulation of intracellular pH, and response to stimuli like oxidative stress and inflammation. GS catalyzes the reaction of ammonia and glutamate to form glutamine, and both the enzyme and its product are involved in the regulation of other enzymes, making them important in various signaling pathways and playing a central role in regulating carbon/nitrogen balance in some parasites (Kumar et al., 2017[97], 2021[96]). In *Trypanosoma cruzi*, for instance, GS is also associated with the ability to escape from the parasitophorous vacuole, allowing it to replicate and infect other cells (Marchese et al., 2018[108]).

In *L. donovani*, this enzyme is expressed in both promastigote and amastigote forms and has been used as a therapeutic target for visceral leishmaniasis, as it is involved in fundamental parasite processes. This ubiquitous protein is engaged in synthesis and recycling processes, allowing the parasite to adapt to various cellular conditions. In *in vitro* and *in vivo* studies by Kumar et al. (2021[96]), it was observed that BALB/c mice infected with GS-knockout *L. donovani* presented a reduction of 96.4 % in parasite burden, and an ~2.4-fold increase in sensitivity to miltefosine *in vitro*. Among the included studies of this present review, Kashif & Subbarao (2023[86]) was the only ones that evaluated this target in *L. major*, and found two promising candidates among 4 inhibitors based on MD simulations and PBSA: chlortalidone, a vasopressin inhibitor used in high blood pressure (binding free energy -294.677 ± 34.571 kJ/mol), and ciprofloxacin, a broad-spectrum fluoroquinolone antibiotic, mainly used in chest and uncomplicated urinary tract infections (binding free energy -19.572 ± 32.555 kJ/mol).

Fluoroquinolones have demonstrated leishmanicidal effects by treating infected macrophages and causing alterations in mitochondrial membrane potentials, as shown in a previous study by Tavares et al. (2019[198]), where the fluoroquinoline derivative (GF1061), in comparison to amphotericin B, was studied *in vitro* and *in vivo* against *L. amazonensis* and *L. infantum* promastigotes and axenic amastigotes, thus affecting the cellular integrity of the parasite and reducing parasitemia in BALB/C mice (P < 0.005) (Tavares et al., 2019[198]).

#### Non-specific nucleoside hydrolase

The non-specific nucleoside hydrolase (NNH) (Figure 6(Fig. 6)) is a class of enzymes found in various organisms, including bacteria, fungi, plants, and animals (but not mammals). It plays a crucial role in breaking down nucleosides into their basic components (e.g. sugars and nitrogenous bases). Since *Leishmania *spp. are obligatory intracellular parasitic protozoa and unable to synthesize purine de novo, they need to salvage bases and nucleosides directly or by cleavage to release nucleobases from the host to sustain their metabolism and replication (Figueroa-Villar and Sales, 2017[57]; Anchau Wegermann et al., 2024[7]; Barazorda-Ccahuana et al., 2024[15]). NNH lacks strict specificity and catalyzes the hydrolysis of the N-ribosyl group of all purine and pyrimidine nucleosides, making it important in a variety of metabolic processes and as a therapeutical target for leishmaniasis (Boitz et al., 2012[26]; Shaposhnikov et al., 2023[185]; Anchau Wegermann et al., 2024[7]).

Among the included studies, Waugh et al. (2014[213]), the only study evaluating NNH interactions from *L. major*, found 16 potential scaffold candidates, such as mannitol, kanamycin, and pitavastatin, which achieved the highest similarity scores to specific ZINC hits. Pitavastatin is an HMG-CoA reductase inhibitor used to lower blood cholesterol levels. This drug and other statins can be evaluated for their potential interference in the sterol pathway for *Leishmania*. Lovastatin, alone or when associated with chromium chloride, induced cytotoxicity mediated by macrophage production of H2O2 against amastigote of *L. donovani*, reducing their intracellular survival (Verma et al., 2017[208]). Apart from nucleoside analogs (Rennó et al., 2012[158]), flavonoids (Nirma et al., 2019[132]), and proanthocyanidins (Casanova et al., 2020[34]) acting as NHs inhibitors, immucillin derivatives were potent anti-amastigotes *in vitro* (Freitas et al., 2015[61]). However, the affinity for specific nucleoside hydrolases should be considered when targeting the salvage pathway (Cui et al., 2001[43]). 

Another candidate drug identified in the included study was paromomycin (similarity score of 0.94 with ZINC compound), an aminoglycoside antibiotic already prescribed for CL. The development of new formulations using micro and nanotechnology, in addition to the association with other antileishmanial drugs, is being studied to increase efficacy in reducing parasitemia and lesions from CL (Dos Santos Matos et al., 2020[49]; Pokharel et al., 2021[148]). Previously, Das et al. (2012[47]) observed *in vitro* that both paromomycin and miltefosine can act on the release of pro-inflammatory molecules, TNF-α and NO, mediated by toll-like receptor 4 (TLR-4) of the host cell, as well as on NF-κB activation, resulting in dose-dependent death of intracellular *L. donovani*. However, the recommendation is to combine these drugs to reduce the potential for antileishmanial drug resistance, especially considering countries like India, where these treatments are known to be cost-effective, particularly in monotherapy. In an *in silico* study by Vacas et al. (2019[202]) with putatively essential Ser/Thr kinase *L. major* (LmjF.22.0810), a lower sensitivity of the parasite to aminoglycosides was also observed, implying the need for further studies evaluating this treatment and its combinations for VL.

#### Arabinono-1-4-lactone oxidase

Arabinono-1,4-lactone oxidase (ALO) (Figure 6(Fig. 6)) is an enzyme involved in the biosynthesis of ascorbate, or vitamin C, by oxidizing D-arabinono-1,4-lactone to D-erythroascorbate. Ascorbic acid is an effective antioxidant, providing protection against oxidative stress, acting as an electron donor for ascorbate-dependent peroxidases (APXs), and also serving as a cofactor in various mammalian metabolic pathways, for instance, biosynthesis of collagen, neurotransmitters, and peptides. These trypanosomatid parasites are also capable of synthesizing ascorbate from common intermediates found in plants and some fungi, such as L-galactono-1,4-lactone and D-arabinono-1,4-lactone (Lee et al., 1999[99]; Kudryashova et al., 2011[94]; Adinehbeigi et al., 2020[3]).

In fungi, although the entire mechanism involving ALO and its function has not been fully elucidated yet, it has been observed that the absence of this enzyme can affect metabolic pathways related to normal physiology, fungal growth, and conidiogenesis (Adinehbeigi et al., 2020[3]; Wu et al., 2022[219]). In *L. donovani*, knockout of ALO reduces growth *in vitro* and *in vivo* infectivity, coupled with increased production of IL-12, INF-γ, and TNF-α from macrophages. Interestingly, ALO also seems to protect amastigotes from macrophage oxidative stress through ascorbate-mediated scavenging (Manhas et al., 2014[107]). For *L. donovani*, Adinehbeigi et al. (2020[3]) identified suramin as the most promising candidate in terms of binding affinity, with the amino acids Leu13, Lys54, Thr116, Cys121, Glu183, and Val184 involved in the interaction between ALO and suramin. Other works confirmed suramin activity against *L. donovani* *in vitro*, and the drug exhibited a ~20-fold selectivity against amastigote forms. *In vitro* experiments using BALB/C mice showed an 84 % reduction in hepatic parasite load after two weeks in a 20 mg/kg/day regimen (Khanra et al., 2018[89], 2020[88]). 

### Potential candidates for repurposing

Although correlating *in vitro* results with efficacy *in vivo* is difficult, Katsuno et al. (2015[87]) suggested an IC_50 _< 10 μM against *L. donovani* intracellular amastigotes as relevant criteria for a hit. Considering the compounds evaluated *in vitro*, fifteen molecules presented an IC_50_ of less than 10 µM against at least one *Leishmania *species: Abemaciclib, bazedoxifene, afatinib, nitazoxanide, perphenazine, rifabutin, luliconazole, dutasteride, chlorhexidine, protriptyline, bortezomib, terconazole, almitrine, midostaurin, and ABT239, indicating promising potency. However, it's important to consider that their antileishmanial activity may not be target-selective. Additionally, variations in assay conditions may lead to divergent results (Moffat et al., 2017[115]). Researchers should consider administration routes, which, in the case of luliconazole, despite its excellent potency, makes it a good candidate only for topical application against CL. Injectable drugs, such as proteasome inhibitor bortezomib, face the same problem as antimonials, AmphB, and pentamidine, making them less innovative. Few of these drugs were evaluated *in vivo* in trypanosomatids. A nanotransferosomal gel containing a combination of nitazoxanide and quercetin reduced the size of CL lesions in BALB/c mice (Bashir et al., 2023[16]). In *T. cruzi-*infected mice, protriptyline had 2.92 times better efficacy than nifurtimox (Kinnamon et al., 1997[91]).

Currently, repurposable drugs, or new combination therapies using current drugs, are under investigation in clinical trials, for instance, Ivermectin, tofacitinib with meglumine antimoniate, intralesional voriconazole, and oral doxycycline. Most trials are focused on cutaneous leishmaniasis. Active clinical trials deposited in ClinicalTrials.gov are presented in Table 2(Tab. 2).

The ability of *Leishmania *to evade immunological response poses an additional challenge to effective treatment (Gupta et al., 2013[69]). For example, pentavalent antimony depends on T-cell activation mediated by INF-γ, for liver parasite clearance in mice (Murray and Delph-Etienne, 2000[127]). The parasite persistence is also observable in the difficulty of achieving sterile immunity, and more pronounced in patients co-infected with HIV suffering from relapses (Sacks and Noben-Trauth, 2002[167]; Hailu et al., 2010[71]; Rodrigues et al., 2016[161]; Takele et al., 2023[197]). Proteins involved in modulating host immune response are interesting drug targets. For instance, cysteine protease B suppresses NF-κB activation, leading to down-regulation of proinflammatory IL-12 production in the infected macrophage (Cameron et al., 2004[31]), also impairing Th1 responses necessary for disease resolution (Buxbaum et al., 2003[30]). Moreover, inhibition of cysteine proteases with compounds such as organotellurium or peptidomimetic aziridines results in a leishmanicidal effect (Salerno Pimentel et al., 2012[170]; Schad et al., 2016[178]). Likewise, rational synthesis targeting cysteine proteases in *Trypanosoma* yielded compounds with nanomolar potency (Ferreira and Andricopulo, 2017[56]).

Interestingly, hamsters infected with *L. donovani* (VL) displayed an important suppression of cytokines involved in leishmaniasis host susceptibility, IL-10, and TGF-β, upon treatment with another tricyclic: imipramine. In addition to parasite inhibition, this immunomodulation seems to favor disease resolution, with a 90 % liver parasite clearance (including an antimony-resistant lineage) for orally treated animals (5 mg/kg/day for 10 days) (Mukherjee et al., 2012[122]), and sterile cure using liposomal imipramine in 4 intermittent doses (10 mg/kg) (Mukherjee et al., 2020[124]). An imipramine-induced HDAC11 overexpression mediates an increase in IL-12/IL-10 ratio (Mukherjee et al., 2014[123]). Moreover, apart from inhibiting TryR, imipramine affects sterol biosynthesis, leading to the accumulation of metabolic intermediates, which could be attributed to SMT inhibition (Garforth et al., 1997[64]; Andrade-Neto et al., 2016[8]). The tricyclic scaffold was associated with leishmanicidal effect by various studies, including drugs such as cyclobenzaprine (Cunha-Júnior et al., 2017[44]), clomipramine (da Silva Rodrigues et al., 2019[45]), amitriptyline (Evans and Croft, 1994[54]), and amitriptyline derivatives (Tonelli et al., 2020[199]), suggesting these compounds could be investigated further.

Considering the investigated literature, two drug classes identified *in silico* and confirmed *in vitro* appear promising for future research: Tricyclic neuroleptics and antidepressants, and azole antifungals. Notably, perphenazine, protriptyline, luliconazole, and terconazole were highlighted in the included studies. These drugs demonstrated high *in vitro* potency against intracellular amastigotes and possess advantageous characteristics, such as high extravascular tissue penetration (Gillman, 2007[66]; Bellmann and Smuszkiewicz, 2017[20]), a relevant characteristic when targeting parasites in the liver, particularly in the case of VL. Additionally, the substantial number of drugs within each class, provides a pool of candidates with different pharmacokinetics, tolerable doses, toxicity profiles, and presumably, antileishmanial activity, without an immediate need to synthesize new analogs. Researchers may take advantage of the available pharmacokinetic data on these drug classes to develop physiologically-based pharmacokinetic models *in silico*. These models could predict whether sufficient concentrations are reached in tissues for effective parasite clearance (Ramisetty et al., 2024[153]) and serve as selection criteria for preclinical studies. More importantly, molecules from these classes should be assessed *in vivo* to account for host-pathogen interactions, especially immunological ones. As demonstrated by Mukherjee et al. (2020[124]), toxicity problems that may arise could be addressed by advanced delivery systems, such as liposomes. Ultimately, this integrative approach of *in silico*, *in vitro*, and *in vivo* validation will be essential in accelerating the identification of effective treatments.

This work aimed to map the literature on drug repurposing supported by *in silico* drug discovery methods against leishmaniasis. Nonetheless, it is important to state the following limitations: (1) The search strategy relied on synonyms of several computational techniques in the title, abstract, or keywords, and studies without the appropriate report of such methods could not be retrieved. Likewise, the selection of studies investigating the repurposing of approved molecules relied on clearly stated information. Additionally, searches were conducted on three online databases, which may not cover every published article on the subject. (2) Studies focused on targets from the host organism instead of the pathogen were not included. (3) Repurposing of biological medicines was not addressed. (4) Given the descriptive condition of a scoping review, quantitative effects could not be statistically summarized.

## Conclusions

The pursuit of safer pharmacotherapeutic alternatives for leishmaniasis treatment has progressed slowly. Repurposing may offer a cost-effective solution around the expensive process of developing an entirely new drug, with *in silico* methodologies playing a role in enhancing efficiency in early steps. This scoping review represented the first comprehensive effort to map the literature on drug repurposing aided by different *in silico* methods against *Leishmania*. Recent studies heavily relied on structure-based drug design (SBDD), particularly molecular docking and dynamics. From the 154 unique drugs evaluated *in silico*, fifteen molecules: Abemaciclib, bazedoxifene, afatinib, nitazoxanide, perphenazine, rifabutin, luliconazole, dutasteride, chlorhexidine, protriptyline, bortezomib, terconazole, almitrine, midostaurin, and ABT239 (~10 %) presented *in vitro* inhibition of *Leishmania *in the lower micromolar range (< 10 µM) against intracellular amastigotes. More importantly, studies introduced new drug alternatives for future research and correctly associated previously known pharmacological classes with *Leishmania*, for example, antifungal azoles and tricyclic antidepressants, the latter producing sterile immunity *in vivo* in liposomal formulation (4 doses of 10 mg/kg). Considering the limited number of agency-approved drugs, future *in silico* research could widen the scope of screened targets (e.g. cysteine proteases, proteasome, and protein kinases) and employ advanced methods, including DFT, multi-target QSAR, and physiologically based pharmacokinetic modeling. Even if the existing set of approved drugs proves insufficient to tackle leishmaniasis, their scaffolds can provide starting points for *in silico* optimization of target affinity and pharmacokinetic improvement.

## Notes

Gustavo Scheiffer and Karime Zeraik Abdalla Domingues contributed equally as first author.

## Declaration

### CRediT authorship contribution statement

GS: Writing - original draft, investigation, conceptualization, formal analysis, visualization. 

KZAD: Writing - original draft, investigation, conceptualization. 

DG: Investigation, writing - review & editing. 

AFC: Writing - review & editing. 

RELL: Writing - review & editing. 

HHLB: Conceptualization, writing - review & editing. 

LMF: Supervision, writing - review & editing. 

RP: Conceptualization, supervision, writing - review & editing.

### Conflict of interest

The authors report no financial or personal conflict of interest regarding this study.

### Funding

This study was partially financed by the Coordenação de Aperfeiçoamento de Pessoal de Nível Superior-Brazil (CAPES) [Finance Code 001] and Conselho Nacional de Desenvolvimento Científico e Tecnológico - Brazil (CNPq) [Proc.: 444941/2023-1]. The funding agencies were not involved in any decisions.

## Supplementary Material

Supplementary information

Supplementary data

## Figures and Tables

**Table 1:**
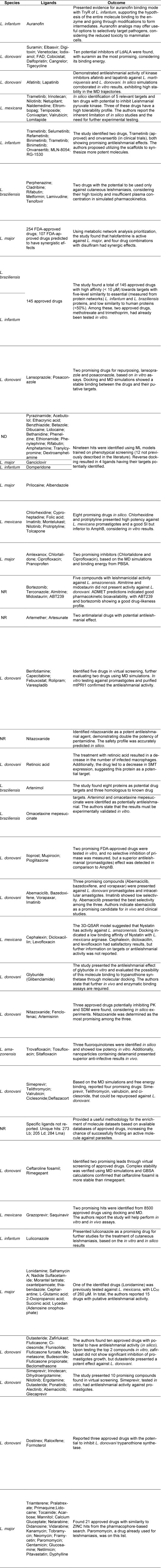
Overview of included studies

**Table 2:**
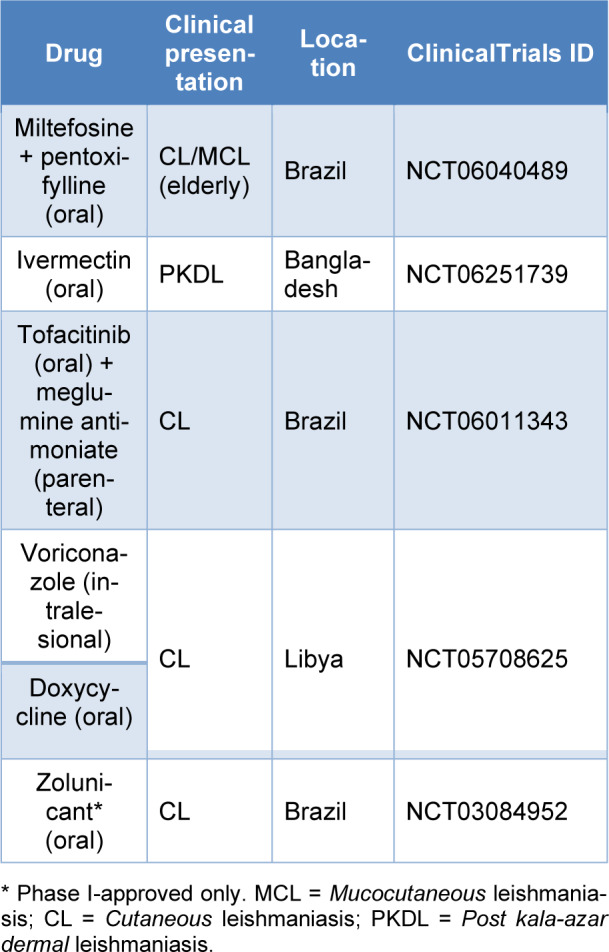
Approved drugs in active clinical trials for leishmaniasis treatment in ClinicalTrials.gov

**Figure 1:**
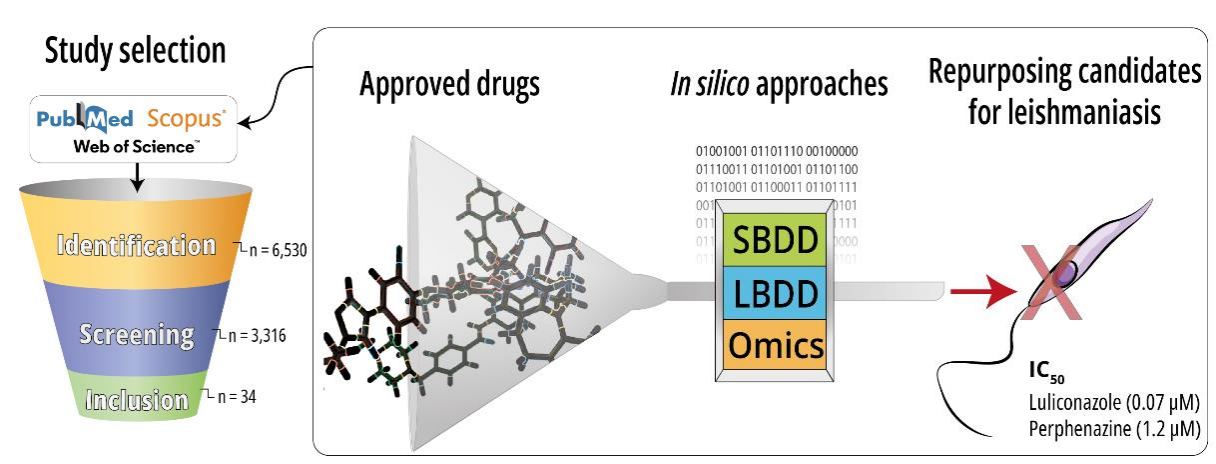
Graphical abstract
