# Supplementary material for: In silico approaches supporting drug repurposing for Leishmaniasis: a scoping review
Source: EXCLI J. 2024 Sep 3;23:1117–69. doi: 10.17179/excli2024-7552 (PMC11484518; doi:10.17179/excli2024-7552)
Supplement: Supplementary information [file EXCLI-23-1117-s-001.pdf]

EXCLI Journal 2024;23:1117-1169 – ISSN 1611-2156
Received: June 24, 2024, accepted: August 08, 2024, published: September 03, 2024

Supplementary information to:

Review article:

IN SILICO APPROACHES SUPPORTING DRUG REPURPOSING FOR LEISHMANIASIS: A SCOPING REVIEW

Gustavo Scheiffer[#], Karime Zeraik Abdalla Domingues[#], Daniela Gorski, Alexandre de Fátima Cobre, Raul Edison Luna Lazo, Helena Hiemisch Lobo Borba, Luana Mota Ferreira, Roberto Pontarolo*

Postgraduate Program in Pharmaceutical Sciences, Department of Pharmacy, Federal University of Paraná, Curitiba 80210-170, Paraná, Brazil

[#] Gustavo Scheiffer and Karime Zeraik Abdalla Domingues contributed equally to this paper.

* **Corresponding author:** Roberto Pontarolo, Departamento de Farmácia - Universidade Federal do Paraná, Av. Prefeito Lothário Meissner 632, Curitiba, PR, Brazil
Zip code 80210-170. Tel. +55 41 3360-4094, E-mail: pontarolo@ufpr.br

https://dx.doi.org/10.17179/excli2024-7552

This is an Open Access article distributed under the terms of the Creative Commons Attribution License (http://creativecommons.org/licenses/by/4.0/).

EXCLI Journal 2024;23:1117-1169 – ISSN 1611-2156
Received: June 24, 2024, accepted: August 08, 2024, published: September 03, 2024

Supplement 1: Search strategies for Scopus, PubMed, and Web of Science databases

Scopus:
(((TITLE-ABS-KEY (leishmania*) OR TITLE-ABS-KEY (leishmaniasis) OR TITLE-ABS-KEY ("antileishmania*") OR TITLE-ABS-KEY ("leishmani*"))) AND (TITLE-ABS-KEY ("computer simulation*") OR TITLE-ABS-KEY ("in silico*") OR (TITLE-ABS-KEY ("bioinformatic*") AND TITLE-ABS-KEY ("drug*")) OR TITLE-ABS-KEY ("ADME*") OR TITLE-ABS-KEY ("CADD") OR (TITLE-ABS-KEY ("Computer Aided*") AND TITLE-ABS-KEY ("Drug Design*")) OR (TITLE-ABS-KEY ("Drug Design*") AND TITLE-ABS-KEY ("Computerized")) OR TITLE-ABS-KEY ("Structure Guide*") OR TITLE-ABS-KEY ("SBDD") OR TITLE-ABS-KEY ("Structure Based*") OR TITLE-ABS-KEY ("LBDD") OR TITLE-ABS-KEY ("Ligand Based*") OR TITLE-ABS-KEY ("fragment based*") OR (TITLE-ABS-KEY ("computer*") AND TITLE-ABS-KEY ("model*")) OR TITLE-ABS-KEY ("docking") OR TITLE-ABS-KEY ("Molecular Dynamic*") OR TITLE-ABS-KEY ("MD Simulation*") OR TITLE-ABS-KEY ("virtual screening*") OR TITLE-ABS-KEY ("free energ*") OR TITLE-ABS-KEY ("binding energ*") OR TITLE-ABS-KEY ("binding affinit*") OR TITLE-ABS-KEY ("*GBSA") OR TITLE-ABS-KEY ("*PBSA") OR TITLE-ABS-KEY ("force field*") OR TITLE-ABS-KEY ("machine learning*") OR TITLE-ABS-KEY ("molecular model*") OR TITLE-ABS-KEY ("pharmacophoric*") OR TITLE-ABS-KEY ("pharmacophore*")))

PubMed:
("Leishmania*"[tiab] OR "Leishmaniasis"[tiab] OR "Leishmaniasis"[MeSH Terms] OR "Antileishmania*"[tiab] OR "Leishmani*"[tiab]) AND ("Computer Simulation"[MeSH Terms] OR "computer simulation*"[tiab] OR "Molecular Docking Simulation"[MeSH Terms] OR "In silico*"[tiab] OR ("bioinformatic*"[tiab] AND "drug*"[tiab]) OR "ADME*"[tiab] OR "CADD"[tiab] OR ("Computer Aided*"[tiab] AND "Drug Design"[tiab]) OR ("Drug Design*"[tiab] AND "Computerized"[tiab]) OR "Structure Guide*"[tiab] OR "SBDD"[tiab] OR "Structure Based*"[tiab] OR "LBDD"[tiab] OR "Ligand Based*"[tiab] OR "fragment based*"[tiab] OR ("computer*"[tiab] AND "model*"[tiab]) OR "Docking"[tiab] OR "Molecular Dynamic*"[tiab] OR "MD Simulation*"[tiab] OR "virtual screening*"[tiab] OR "free energ*"[tiab] OR "binding energ*"[tiab] OR "binding affinit*"[tiab] OR "*GBSA"[tiab] OR "*PBSA"[tiab] OR "force field*"[tiab] OR "machine learning*"[tiab] OR "Molecular Model*"[tiab] OR "pharmacophoric*"[tiab] OR "pharmacophore*"[tiab])

Web of Science:
(((TS=(Leishmania*) OR TS=(leishmaniasis) OR TS=("antileishmania*") OR TS=("leishmani*"))) AND (TS=("computer simulation*") OR TS=("in silico*") OR (TS=("bioinfomatic*") AND TS=("drug*")) OR TS=("ADME*") OR TS=("CADD") OR (TS=("Computer Aided*") AND TS=("Drug Design*")) OR (TS=("Drug Design*") AND TS=("Computerized")) OR TS=("Structure Guide*") OR TS=("SBDD") OR TS=("Structure Based*") OR TS=("LBDD") OR TS=("Ligand Based*") OR TS=("fragment based*") OR (TS=("computer*") AND TS=("model*")) OR TS=("docking") OR TS=("Molecular Dynamic*") OR TS=("MD Simulation*") OR TS=("virtual screening*") OR TS=("free energ*") OR TS=("binding energ*") OR TS=("binding affinit*") OR TS=("*GBSA") OR TS=("*PBSA") OR TS=("force field*") OR TS=("machine learning*") OR TS=("molecular model*") OR TS=("pharmacophoric*") OR TS=("pharmacophore*")))

EXCLI Journal 2024;23:1117-1169 – ISSN 1611-2156
Received: June 24, 2024, accepted: August 08, 2024, published: September 03, 2024

Supplement 2: Data Extraction Guide

Review objective: To assess the methodologies, results and outcomes related to *in silico* leishmaniasis drug repurposing.

Review question: "What are the main methodologies, results, and outcomes presented on *in silico* studies of anti-leishmanial drug repurposing?"

**INCLUSION/EXCLUSION CRITERIA**	
**Population**	N/A
**Concept**	Utilization of *in silico* methods for repurposing approved drugs against leishmaniasis.
**Context**	**Methodology** (e.g.: Software, parameters, targets, databases, techniques); **Results** (e.g.: ligand-target interactions, structural/chemical info about the ligands) and **outcomes** (e.g.: how many promising candidates were detected, lack/presence of *in vitro* confirmation) presented on the studies. Potential research gaps are to be identified and discussed.
**Type of evidence source**	**Primary studies found in databases,** involving the usage of **computational (*in silico*) methods for anti-leishmanial drug repurposing**, including the following study designs: solely *in silico* studies, those with additional *in vitro* and/or *in vivo* testing and *in vitro/in vivo* studies clearly mentioning a previously published *in silico* step.<br><br>Studies **not** to be included:<br>• Experimental, not-clinically studied drugs (at least Phase I)<br>• Genomic, metabolic, proteomic, and other studies not involving drugs<br>• Studies involving simple putative targets (not testing a molecule against it)<br>• Studies aiming to identify or test an immunogenic target (vaccine development)<br>• Studies not directly investigating anti-leishmanial compounds (other NTDs)<br>• Studies focused on vectors (*Phlebotominae*)

EXCLI Journal 2024;23:1117-1169 – ISSN 1611-2156
Received: June 24, 2024, accepted: August 08, 2024, published: September 03, 2024

<table>
<tr><th colspan="3">SOURCE INFORMATION</th></tr>
<tr><th colspan="2">Category</th><th>Description/examples</th></tr>
<tr><td colspan="2">Citation details</td><td>• First author et al. (year)
• Title
• Country
• DOI</td></tr>
<tr><td colspan="2">Article objective/aims</td><td>Brief and concise description of the main objective(s) of the study. E.g.:
• “Performing a virtual screening of 1500 FDA-approved compounds against the enzyme X”
• “Docking and MD simulations analyzing a previously identified inhibitor of enzyme X”</td></tr>
<tr><td colspan="2">Methodology
(in silico/in vitro)</td><td>• SBDD, LBDD or Omics (Adapt accordingly)
• Virtual screening (Y/N)
    • Software
    • Docking, ligand-based or other (put additional details in the specific category)
    • Number of molecules, databases (e.g., ZINC), criteria for selecting the databases (FDA-approved, Natural Products)
• Docking
    • Software
    • Parameters
    • Docking region
• Cutoff criteria for hits
    • Binding energy, docking score, stability during MD
• Further validation of hits by MD (Y/N)
    • MD software/version
    • Simulation time, replicates, force field, solvent model
    • Parameters (solvent implicit or explicit, temperature, any preparation steps).
• Pharmacophoric model (Y/N)
    • Number of molecules used to build the model, software, 2D/3D
    • Software
• Machine-learning model (Y/N)
    • Number of molecules, datasets
    • Algorithm(s)
    • AUC, accuracy
• ADMET prediction (y/s)
    • Criteria used for incl/excl.
    • Software/website</td></tr>
<tr><td rowspan="2">DATA</td><td>Results/
Key findings</td><td>• Number of promising hits
• Relevant interactions and respective amino acids residues
• Binding pockets
• Competitive or allosteric modulator
• MD results
    • Stable or unstable complexes
• Best ligands</td></tr>
<tr><td>Ligand/s</td><td>• Name (used by the authors)
• Class (or scaffold)
• Numeric results of top hits (from docking or ligand-based techniques)
    • Scores, binding energies
• In vitro assay (Y/N):</td></tr>
</table>

EXCLI Journal 2024;23:1117-1169 – ISSN 1611-2156
Received: June 24, 2024, accepted: August 08, 2024, published: September 03, 2024

		**Describe the main points:**<br>• Species of Leishmania tested<br>• Purified protein/enzymatic inhibition assay (Y/N)<br>• Infected macrophages, axenic amastigotes, promastigotes<br>• Potency (IC_{50}/EC_{50}) for each species<br>• xx.x µM<br>• Methodology used for viability assay (e.g.: MTT)<br>• Selectivity index (SI)
	**Target/s (if appl.)**	• Name (use unified naming)<br>• PDB ID Entry Code (if. appl.) or structure generation method (e.g. homology modeling)<br>• Involved metabolic pathway
**Outcome/s**		Describe the main conclusion of the article, if the outcome is favorable or unfavorable for supporting the discovery of an anti-leishmanial drug.<br>• "*In silico* research resulted in the discovery of 3 compounds with *in vitro* anti-Leishmanial activity."<br>• "The authors found a low SI."<br>• "The study did not reach the *in vivo* phase due to the observed toxicity"<br>• "*In silico* hits exhibited unfavorable predicted ADMET properties"<br>• Authors comments about next steps (*in vitro/in vivo*)

Important - Screening process (Rayyan):

- **Report exclusion reasons for each article**
- Label articles (even those excluded, but especially for included articles) for future retrieval and subgrouping:
    - Use relevant labels: Target name, main methods (docking, MD, QSAR, ADMET, pharmacophore), chemical classification of ligands, *in vitro*.

Supplement 3: Exclusion reasons

Reason	Title	Year	Journal	Authors	Publisher	DOI
Conference paper	Virtual Screening of Leishmanial Pyridoxal Kinase Enzyme Inhibitors by Repurposed Anti-Trypanosomal Libraries Reveals Two Core Scaffolds	2021	ChemRxiv. 2020	*Alfadhel, S.*	Colegio de Farmaceuticos de la Provincia de Buenos Aires	10.26434/chemrxiv.13077395.v1
Non-approved	Antiprotozoan lead discovery by aligning dry and wet screening: Prediction, synthesis, and biological assay of novel quinoxalinones	2014	Bioorganic & Medicinal Chemistry	*Martins Alho MA, Marrero-Ponce Y, Barigye SJ, Meneses-Marcel A, Machado Tugores Y, Montero-Torres A, Gómez-Barrio A, Nogal JJ, García-Sánchez RN, Vega MC, Rolón M, Martínez-Fernández AR, Escario JA, Pérez-Giménez F, Garcia-Domenech R, Rivera N, Mondragón R, Mondragón M, Ibarra-Velarde F, Lopez-Arencibia A, Martín-Navarro C, Lorenzo-Morales J, Cabrera-Serra MG, Piñero J, Tytgat J, Chicharro R, Arán VJ.*	Elsevier	10.1016/j.bmc.2014.01.036
Non-roman	Identification of Agents with Potential Leishmania Malate Dehydrogenase Inhibitor Activity: A Proteomic and Molecular Docking Approach	2022	Journal of Mazandaran University of Medical Sciences	*Amiri-Dashatan N, Koushki M, Ashrafmansouri M, Ahmadi N.*	Mazandaran University of Medical Sciences	
Non-approved	Specific Human ATR and ATM Inhibitors Modulate Single Strand DNA Formation in Leishmania major Exposed to Oxidative Agent	2022	Frontiers in Cellular and Infection Microbiology	*da Silva RB, Bertoldo WDR, Naves LL, de Vito FB, Damasceno JD, Tosi LRO, Machado CR, Pedrosa AL. and Tosi, L.R.O. and Machado, C.R. and Pedrosa, A.L.*	Frontiers Media S.A.	10.3389/fcimb.2021.802613
Non-approved	Selective human inhibitors of ATR and ATM render Leishmania major promastigotes sensitive to oxidative damage	2018	PLoS One	*da Silva RB, Machado CR, Rodrigues ARA, Pedrosa AL.*	PLOS ONE	10.1371/journal.pone.0205033

Compounds tested in silico aren't approved	Virtual Screening Identification of Nonfolate Compounds, Including a CNS Drug, as Antiparasitic Agents Inhibiting Pteridine Reductase	2011	Journal of Medicinal Chemistry	*Ferrari S, Morandi F, Motiejunas D, Nerini E, Henrich S, Luciani R, Venturelli A, Lazzari S, Calò S, Gupta S, Hannaert V, Michels PA, Wade RC, Costi MP.*	ACS Publications	10.1021/jm1010572
Non-approved	Repurposing of conformationally-restricted cyclopentane-based AKT–inhibitors leads to discovery of potential and more selective antileishmanial agents than miltefosine	2023	Bioorganic Chemistry	*Hassan AHE, Alam MM, Phan TN, Baek KH, Lee H, Cho SB, Lee CH, Kim YJ, No JH, Lee YS.*	Academic Press Inc.	10.1016/j.bioorg.2023.106890
Non-approved	Rational repurposing, synthesis, in vitro and in silico studies of chromone-peptidyl hybrids as potential agents against Leishmania donovani	2023	Journal of Enzyme Inhibition and Medicinal Chemistry	*Hassan AHE, Bayoumi WA, El-Sayed SM, Phan TN, Kim YJ, Lee CH, Cho SB, Oh T, Ham G, Mahmoud K, No JH, Lee YS.*	Taylor & Francis	10.1080/14756366.2023.2229071
Non-approved	Bestatin analogs-4-quinolinone hybrids as antileishmanial hits: Design, repurposing rational, synthesis, in vitro and in silico studies	2023	European Journal of Medicinal Chemistry	*Hassan AHE, Mahmoud K, Phan TN, Shaldam MA, Lee CH, Kim YJ, Cho SB, Bayoumi WA, El-Sayed SM, Choi Y, Moon S, No JH, Lee YS.*	Elsevier	10.1016/j.ejmech.2023.115211
Non-approved	Design, Rational Repurposing, Synthesis, In Vitro Evaluation, Homology Modeling and In Silico Study of Sulfuretin Analogs as Potential Antileishmanial Hit Compounds	2022	Pharmaceuticals	*Hassan AHE, Phan TN, Choi Y, Moon S, No JH, Lee YS.*	MDPI	10.3390/ph15091058
Non-approved	Design, synthesis, and repurposing of O6-aminoalkyl-sulfuretin analogs towards discovery of potential lead	2023	European Journal of Medicinal Chemistry	*Hassan AHE, Phan TN, Moon S, Lee CH, Kim YJ, Bin Cho S, El-Sayed SM, Choi Y, No JH, Lee YS.*	Elsevier	10.1016/j.ejmech.2023.115256

	compounds as antileishmanial agents					
Non-approved	Pyrrolidine-based 3-deoxysphingosylphosphorylcholine analogs as possible candidates against neglected tropical diseases (NTDs): identification of hit compounds towards development of potential treatment of Leishmania donovani	2021	Journal of Enzyme Inhibition and Medicinal Chemistry	*Hassan AHE, Phan TN, Yoon S, Lee CJ, Jeon HR, Kim SH, No JH, Lee YS.*	Taylor & Francis	10.1080/14756366.2021.1969385
Non-approved	Repurposing azole antifungals into antileishmanials: Novel 3-triazolylflavanones with promising in vitro antileishmanial activity against Leishmania major	2019	Parasitology International	*Keighobadi M, Emami S, Fakhar M, Shokri A, Mirzaei H, Hosseini Teshnizi S.*	Elsevier	10.1016/j.parint.2018.12.006
Conference paper	Drug Repurposing Using Knowledge Graph Embeddings with a Focus on Vector-Borne Diseases: A Model Comparison	2023	Communications in Computer and Information Science	*López Yse D, Torres D.*	Springer Science and Business Media Deutschland GmbH	10.1007/978-3-031-40942-4_8
Non-approved	Virtual and experimental screening of phenylfuranchalcones as potential anti-Leishmania candidates	2019	Journal of Molecular Graphics & Modelling	*Ochoa R, García E, Robledo SM, Cardona W.*	Elsevier	10.1016/j.jmgm.2019.06.015
Conference paper	Prediction of potential kinase inhibitors in Leishmania spp. through a machine learning and molecular docking approach	2014	Advances in Intelligent Systems and Computing	*Ochoa R, Davies M, Flórez A, Espinosa J, Muskus C.*	Springer Verlag	10.1007/978-3-319-01568-2_9

Non-approved	Febrifugine dihydrochloride as a new oral chemotherapeutic agent against visceral leishmaniasis infection	2022	Experimental Parasitology	*Pandey RK, Ojha R, Devender M, Sebastian P, Namdeo M, Kumbhar BV, Sundar S, Maurya R, Prajapati VK.*	Elsevier	10.1016/j.exppara.2022.108250
Macromolecule	In silico molecular modeling and docking studies on the Leishmania mitochondrial iron transporter-1 (LMIT1)	2020	Comparative Clinical Pathology	*Pasandideh R, Dadmanesh M, Khalili S, Mard-Soltani M, Ghorban K.*	Springer	10.1007/s00580-019-03033-7
Resistance	Three different mutations in the DNA topoisomerase 1B in Leishmania infantum contribute to resistance to antitumor drug topotecan	2021	Parasites and Vectors	*Rosa-Teijeiro C, Wagner V, Corbeil A, d'Annessa I, Leprohon P, do Monte-Neto RL, Fernandez-Prada C.*	BioMed Central Ltd	10.1186/s13071-021-04947-4
Methodology conflicts	In-Silico Identification of the Best Compound Against Leishmania infantum: High Throughput Screening of All FDA Approved Drugs	2019	Turkiye Parazitoloji Dergisi	*Saki J, Shadnoush F, Arjmand R, Rahim F.*	Galenos Publishing House	10.4274/tpd.galenos.2019.6290
Non-approved	Repurposing the Trypanosomatidic GSK Kinetobox for the Inhibition of Parasitic Pteridine and Dihydrofolate Reductases	2021	Pharmaceuticals	*Santucci M, Luciani R, Gianquinto E, Pozzi C, Pisa FD, Dello Iacono L, Landi G, Tagliazucchi L, Mangani S, Spyrakis F, Costi MP.*	MDPI	10.3390/ph14121246
Previous in silico step could not be found	Study of Ethinyl Estradiol Activity Against Promastigotes, Axenic and Macrophage-Dwelling Amastigotes of Leishmania infantum by Using Atomic Force Microscopy and Methyl Thiazolyl Tetrazolium Methods	2019	Jundishapur Journal of Microbiology	*Shadnoush F, Arjmand R, Rahim F, Saki J.*	Brieflands	10.5812/jjm.90857

Non-approved	Unveiling six potent and highly selective antileishmanial agents via the open source compound collection 'Pathogen Box' against antimony-sensitive and -resistant Leishmania braziliensis	2021	Biomedicine & Pharmacotherapy	*Silva JAS, Tunes LG, Coimbra RS, Ascher DB, Pires DEV, Monte-Neto RL.*		10.1016/j.biopha.2020.111049
Compounds tested in silico arent approved	Targeting Lysine Deacetylases (KDACs) in Parasites	2015	PloS Neglected Tropical Diseases	*Wang Q, Rosa BA, Nare B, Powell K, Valente S, Rotili D, Mai A, Marshall GR, Mitreva M.*	PLOS ONE	10.1371/journal.pntd.0004026
Non-approved	Design, Synthesis, and Repurposing of Rosmarinic Acid-β-Amino-α-Ketoamide Hybrids as Antileishmanial Agents	2023	Pharmaceuticals	Hassan AHE, Bayoumi WA, El-Sayed SM, Phan T-N, Oh T, Ham G, Mahmoud K, No JH, Lee YS.	MDPI	10.3390/ph16111594
Non-approved	Rational repurposing; synthesis; in vitro and in silico studies of chromone-peptidyl hybrids as potential agents against Leishmania donovani	2023	Journal of Enzyme Inhibition and Medicinal Chemistry	Hassan AHE, Bayoumi WA, El-Sayed SM, Phan TN, Kim YJ, Lee CH, Cho SB, Oh T, Ham G, Mahmoud K, No JH, Lee YS.	Taylor & Francis	10.1080/14756366.2023.2229071

EXCLI Journal 2024;23:1117-1169 – ISSN 1611-2156
Received: June 24, 2024, accepted: August 08, 2024, published: September 03, 2024

Supplement 4: will be found as separate Supplementary data in xlsx format attached to the article.

EXCLI Journal 2024;23:1117-1169 – ISSN 1611-2156
Received: June 24, 2024, accepted: August 08, 2024, published: September 03, 2024

Supplement 5: Binding energies for target-ligand complexes

Citation	Name	Ligands	Binding free energy
Abhishek et al. (2019)	**Trypanothione reductase**	Auranofin	−36.72 kcal/mol (GBSA)
Adinehbeigi et al. (2019)	**Arabinono-1, 4-lactone oxidase**	Suramin	-28.9 kJ/mol (Vina)
		Elbasvir	-27.9 kJ/mol (Vina)
		Digitoxin	-24.7 kJ/mol (Vina)
		Venetoclax	-23.2 kJ/mol (Vina)
		Iodixanol	-22.6 kJ/mol (Vina)
		FAD	-22.1 kJ/mol (Vina)
		Cobicistat	-21.4 kJ/mol (Vina)
		Dalfopristin	-20.5 kJ/mol (Vina)
		Cangrelor	-19.8 kJ/mol (Vina)
		Tigecycline	-19.7 kJ/mol (Vina)
Aiebchun et al. (2023)	**Mitogen-activated protein kinase 3***	Afatinib	~-100 kcal/mol*** (GBSA)
		Lapatinib	~-100 kcal/mol*** (GBSA)
Amiri-Dashatan et al. (2021)	**Pyruvate kinase**	Trametinib	-10.4 kcal/mol (Vina)
		Irinotecan	-10.3 kcal/mol (Vina)
		Nilotinib	-10.1 kcal/mol (Vina)
		Netupitant	-10.1 kcal/mol (Vina)
		Naldemedine	-10.1 kcal/mol (Vina)
		Eltrombopag	-10.0 kcal/mol (Vina)
		Teniposide	-9.9 kcal/mol (Vina)
		Conivaptan	-9.9 kcal/mol (Vina)
		Valrubicin	-9.9 kcal/mol (Vina)
		Lomitapide	-9.8 kcal/mol (Vina)
Gupta et al. (2022)	**Calcium motive P-type ATPase**	Lansoprazole	-63.58 kcal/mol (R)<br>-41.09 kcal/mol (S)<br>(GBSA)
	**Calcium-transporting ATPase**		-60.85 kcal/mol (R)<br>-58.63 kcal/mol

EXCLI Journal 2024;23:1117-1169 – ISSN 1611-2156
Received: June 24, 2024, accepted: August 08, 2024, published: September 03, 2024

			(S)<br>(GBSA)
	**P-type ATPase**		-33.04 kcal/mol<br>(R)<br>-63.66 kcal/mol<br>(S)<br>(GBSA)
Harigua-Souiai et al. (2022)	**MAP Kinase 10**	Prilocaine	–10.3 kcal/mol<br>(Vina)
	**N-myristoyltransferase**	Albendazole	–10.2 kcal/mol<br>(Vina)
	**Pteridine reductase 1**	Ganciclovir	–10.1 kcal/mol<br>(Vina)
	**Trypanothione reductase**	Domperidone	–12.2 kcal/mol<br>(Vina)
Kashif and Subbarao (2023)	**Glutamine synthetase**	Amlexanox	-141.843 kJ/mol<br>(PBSA)
		Chlortalidone	-294.677 kJ/mol<br>(PBSA)
		Pranoprofen	-105.079 kJ/mol<br>(PBSA)
		Ciprofloxacin	-19.572 kJ/mol<br>(PBSA)
Prakash et al. (2023)	**Sterol 24-C-methyltransfer-ase**	Adapalene	-8.3 kcal/mol<br>(Vina)
		Retinoic acid	-9.9 kcal/mol<br>(Vina)
Prava and Pan (2022)	**Eukaryotic translation initia-tion factor 3 subunit 8 (eIF3)***	Artenimol	-29.749 kcal/mol<br>(Glide energy)
	**Ribosomal protein L2***	Omacetaxine mepesuccinate	-40.706 kcal/mol<br>(Glide energy)
Rai et al. (2022)	**Primase**	Iloprost	-122.60 kcal/mol<br>(GBSA)
		Mupirocin	-112.86 kcal/mol<br>(GBSA)
		Pioglitazone	-88.26 kcal/mol<br>(GBSA)
Ranjan and Dubey (2023)	**Citrate synthase**	Abemaciclib	−203.47 ± 137.75 kcal/mol<br>(PBSA)
		Glimepiride	−41.02 ± 116.36 kcal/mol<br>(PBSA)
		Bazedoxifene	−171.34 ± 193.2 kcal/mol<br>(PBSA)
		Vorapaxar	−221.68 ± 11.95 kcal/mol<br>(PBSA)
		Imatinib	−47.87 ± 42.69 kcal/mol<br>(PBSA)
Rub et al. (2019)	**Trypanothione Synthetase**	Glyburide (Glibenclamide)	-7.6 kcal/mol<br>(Vina)
Saha et al. (2023)	**Pyridoxal kinase**	Nitazoxanide	−100.71 ± 22.01 kJ/mol<br>(PBSA)

		Fenclofenac	−73.23 ± 30.80 kJ/mol (PBSA)
		Artemisinin	−101.79 ± 18.97 kJ/mol (PBSA)
	**Sterol 14 alpha-demethylase**	Nitazoxanide	-175.609 ± 12.64 kJ/mol (PBSA)
		Fenclofenac	−131.93 ± 12.73 kJ/mol (PBSA)
		Artemisinin	−125.25 ± 12.91 kJ/mol (PBSA)
Sarma et al. (2023)	**Glutathione synthetase**	Simeprevir	-89.21 ± 9.36 kcal/mol (PBSA)
		Telithromycin	-45.34 ± 4.42 kcal/mol (PBSA)
		Valrubicin	-37.04 ± 4.62 kcal/mol (PBSA)
		Deflazacort	−19.47±14.96 kcal/mol (GBSA)
		Ciclesonide	−22.11 ± 0.42 kcal/mol (GBSA)
Sheikh et al. (2023a)	**Ornithine decarboxylase**	Ceftaroline fosamil	-91.838 kcal/mol (GBSA)
		Rimegepant	-44.717 kcal/mol (GBSA)
Sheikh et al. (2023b)	**Phosphomannomutase**	Grazoprevir	-8.5 kcal/mol (Vina) -10.137 (Glide gscore)
		Saquinavir	-8.9 kcal/mol (Vina) -10.158 (Glide gscore)
Shokri et al. (2018)	**Sterol 14 alpha-demethylase**	Luliconazole	-8.05 kJ/mol (AutoDock 4.2)
Tabrez et al. (2021a)	**Sterol 14 alpha-demethylase**	Dutasteride	-11.7 kcal/mol (Vina)
		Zafirlukast	-11.7 kcal/mol (Vina)
		Fluticasone	-11.6 kcal/mol (Vina)
		Ciclesonide	-11.6 kcal/mol (Vina)
		Flunisolide	-11.5 kcal/mol (Vina)
		Fluticasone furoate	-11.5 kcal/mol (Vina)
		Mometasone	-11.4 kcal/mol (Vina)

EXCLI Journal 2024;23:1117-1169 – ISSN 1611-2156
Received: June 24, 2024, accepted: August 08, 2024, published: September 03, 2024

		Budesonide	-11.4 kcal/mol (Vina)
		Fluticasone propionate	-11.3 kcal/mol (Vina)
		Beclomethasone	-11.2 kcal/mol (Vina)
Tabrez et al. (2021b)	**Sterol 24-C-methyltransferase**	Simeprevir	-10.6 kcal/mol (Vina)
		Irinotecan	-10.5 kcal/mol (Vina)
		Dihydroergotamine	-10.5 kcal/mol (Vina)
		Nilotinib	-10.3 kcal/mol (Vina)
		Ergotamine	-10.3 kcal/mol (Vina)
		Dutasteride	-10.2 kcal/mol (Vina)
		Ponatinib	-10.1 kcal/mol (Vina)
		Alectinib	-10.1 kcal/mol (Vina)
		Abemaciclib	-10.1 kcal/mol (Vina)
		Glecaprevir	-10.1 kcal/mol (Vina)
Juarez-Saldivar et al. (2024)	**Triosephosphate isomerase**	Chlorhexidine	-8.9 kcal/mol (Vina)
		Cyproheptadine	-8.2 kcal/mol (Vina)
		Folic acid	-7.6 kcal/mol (Vina)
		Imatinib	-8.2 kcal/mol (Vina)
		Montelukast	-7.6 kcal/mol (Vina)
		Nilotinib	-7.6 kcal/mol (Vina)
		Protriptyline	-7.5 kcal/mol (Vina)
		Tolcapone	-7.8 kcal/mol (Vina)
Nath et al. (2024)	**Mitochondrial DNA primase**	Benfotiamine	-69.79 ± 11.49 kcal/mol (GBSA)
		Capecitabine	-39.39 ± 8.21 kcal/mol (GBSA)
		Febuxostat	-7.1 kcal/mol (Vina)
		Rolipram	-7.5 kcal/mol (Vina)
		Varespladib	-8.0 kcal/mol (Vina)
Rashid et al. (2024)	**Arginase**	Cephalexin	-91.186 kcal/mol<br>-93.8321 kcal/mol<br>-94.2872 kcal/mol<br>(Moldock)

EXCLI Journal 2024;23:1117-1169 – ISSN 1611-2156
Received: June 24, 2024, accepted: August 08, 2024, published: September 03, 2024

		Dicloxacillin	-124.112 kcal/mol<br>-121.28 kcal/mol<br>-115.148 kcal/mol<br>(Moldock)
		Levofloxacin	-75.5227 kcal/mol<br>-98.7043 kcal/mol<br>-80.371 kcal/mol<br>(Moldock)
Santamaría-Aguirre et al. (2023)	**Topoisomerase II**	Trovafloxacin	NR
		Tosufloxacin	
		Sitafloxacin	
Vemula et al. (2024)	**Trypanothione synthetase**	Cabergoline	-75.5 ±9.88 kcal/mol (GBSA)
		Raloxifene	-89.5 ±14.27 kcal/mol (GBSA)
		Formoterol	-51.89 ±6.59 kcal/mol (GBSA)

NR = Not reported; ***Approximate value

EXCLI Journal 2024;23:1117-1169 – ISSN 1611-2156
Received: June 24, 2024, accepted: August 08, 2024, published: September 03, 2024

Supplement 6: Main Targets

Name	Structure ID	UniProt Entry	Subcellular localization (Gene Ontology)
Arabinono-1-4-lactone oxidase	AF-C8CCV9-F1 (Ld)[1]	C8CCV9	Glycosome<br>Membrane
Citrate synthase	AF-E9BDQ0-F1 (Ld)[1]	E9BDQ0	NA
DNA primase	AF-Q4QBV6-F1 (Lm)[1]	Q4QBV6	alpha DNA polymerase:primase complex<br>ciliary plasm<br>cytoplasm<br>nucleoplasm
Glutamine synthetase	AF-Q4QJ42-F1 (Lm)[1]	Q4QJ42	Cytoplasm
Glycerol-3-phosphate dehydrogenase	AF-Q4QHG4-F1 (Lm)[1]	Q4QHG4	Cytosol<br>Glycerol-3-phosphate dehydrogenase complex<br>Glycosome
Nonspecific nucleoside hydrolase	AF-Q4QDN1-F1 (Lm)[1]	Q4QDN1	Cytosol
Pyruvate kinase	3PP7 (Lmex)[2]	Q27686	NA
Sterol 14-alpha demethylase	3L4D (Li)[2]	A2TEF2	NA
Sterol 24-C-methyltransferase	AF-Q4Q1I3-F1 (Lm)[1]	Q4Q1I3	Cytoplasm<br>Endoplasmic reticulum
Trypanothione reductase	2JK6 (Li)[2]	A4HSF7	Cytoplasm
Trypanothione synthetase	2VOB (Lm)[2]	Q711P7	Cytoplasm

[1]AlphaFold structure ID; [2]PDB ID; NA = Not available
